# A Standardized Reference Data Set for Vertebrate Taxon Name Resolution

**DOI:** 10.1371/journal.pone.0146894

**Published:** 2016-01-13

**Authors:** Paula F. Zermoglio, Robert P. Guralnick, John R. Wieczorek

**Affiliations:** 1 Departamento de Ecología, Genética y Evolución, Instituto IEGEBA (CONICET-UBA), Facultad de Ciencias Exactas y Naturales, Universidad de Buenos Aires, Buenos Aires, Argentina; 2 Institut de Recherche sur la Biologie de l’Insecte, UMR 7261 CNRS, Université François Rabelais, Tours, France; 3 University of Florida Museum of Natural History, University of Florida at Gainesville, Gainesville, Florida, United States of America; 4 Museum of Vertebrate Zoology, University of California, Berkeley, California, United States of America; Smithsonian Institution, UNITED STATES

## Abstract

Taxonomic names associated with digitized biocollections labels have flooded into repositories such as GBIF, iDigBio and VertNet. The names on these labels are often misspelled, out of date, or present other problems, as they were often captured only once during accessioning of specimens, or have a history of label changes without clear provenance. Before records are reliably usable in research, it is critical that these issues be addressed. However, still missing is an assessment of the scope of the problem, the effort needed to solve it, and a way to improve effectiveness of tools developed to aid the process. We present a carefully human-vetted analysis of 1000 verbatim scientific names taken at random from those published via the data aggregator VertNet, providing the first rigorously reviewed, reference validation data set. In addition to characterizing formatting problems, human vetting focused on detecting misspelling, synonymy, and the incorrect use of Darwin Core. Our results reveal a sobering view of the challenge ahead, as less than 47% of name strings were found to be currently valid. More optimistically, nearly 97% of name combinations could be resolved to a currently valid name, suggesting that computer-aided approaches may provide feasible means to improve digitized content. Finally, we associated names back to biocollections records and fit logistic models to test potential drivers of issues. A set of candidate variables (geographic region, year collected, higher-level clade, and the institutional digitally accessible data volume) and their 2-way interactions all predict the probability of records having taxon name issues, based on model selection approaches. We strongly encourage further experiments to use this reference data set as a means to compare automated or computer-aided taxon name tools for their ability to resolve and improve the existing wealth of legacy data.

## Introduction

### Taxonomic names in digitized species occurrence data sets are unreliable

Digitized biocollections have already been shown to provide critical baseline data that can inform biodiversity assessment and trend-analysis [1,2]. These data, while important and valuable, must still be evaluated for fitness for use [3]. Recent work has focused on geospatial issues and their impacts on analyses [4,5]. Equally important, and less often performed, are assessments of taxonomic issues [6–8]. Since correct taxon names associated with primary occurrence data are essential for downstream uses in biodiversity studies [9], issues with taxon names strongly limit, the utility of digitized records.

Specimens bear taxonomic names through the act of identification. The output of this process results in a specimen bearing a taxon name [10,11]. Unfortunately, the process of writing those names onto specimen labels or ledgers is neither infallible nor is it usually done as often as is needed to keep names current. Furthermore, names that are handwritten on labels or ledgers associated with specimens must be interpreted and transcribed when digitized. Content on the original labels is prone to human error, such as misspelling, while digitization into fields in collection databases may introduce further errors. When mobilized more broadly, those original data fields may need to be further rationalized to community standards, such as Darwin Core [12,13], before becoming part of the digitally accessible information [14] provisioned by aggregators such as the Global Biodiversity Information Facility (GBIF) [15]. Given all these distinct steps from labels to online accessible data, the end result is that raw digitized taxon name content cannot simply be used as-is without further scrutiny [8,16].

The challenge of improving taxonomic content in legacy data has not gone unaddressed. Many long-standing and new efforts to compile taxonomic changes across different taxonomic groups and register new names have become part of the digital era. These digital scientific name banks can be leveraged for detection and correction of taxon name issues by the scientific community. Examples of taxon- or biome-specific name databases, some of which also serve as registries, include The Plant List [17], Index Fungorum [18], the World Spider Catalog [19], Avibase [20,21], FishBase and Catalog of Fishes [22,23], World Register of Marine Species [24,25], The Reptile Database [26], AmphibiaWeb [27], Mammal Species of the World [28,29], among many others. Broader initiatives that compile these critical resources and provide all-taxon views include the Global Names Architecture [9], the Integrated Taxonomic Information System [30] and the Catalogue of Life (CoL) [31].

The Global Biodiversity Information Facility provides a taxonomic backbone service [32], which reconciles taxonomic information contained in its species occurrence records against a GBIF-assembled set of checklists containing many of the ones listed above. Other aggregators, such as the Ocean Biogeographic Information System (OBIS), have their own taxonomic authority files [33]. Gaiji et al. [34] showed that 9.15% of names found associated with approximately 25 million occurrence records in GBIF (7.8% of the records accessible in 2013) could not be resolved to an existing name in one of its checklists. When a user searches for species occurrence records, GBIF leverages this taxonomic backbone to ensure that the search returns relevant records even if the name does not exactly match the entry or is out of date. They also match synonyms using the taxonomic backbone and provide those records in the same download if a valid name is given in the search criteria. Finally, if one searches on a synonym, GBIF provides the records for that synonym only, but also suggests the valid name prominently in the search interface. GBIF also provides the best-guess valid scientific name for records in all occurrence downloads. While GBIF does not change the original content at the source, nor at the point of data publication, their services help users find the content they ultimately seek. This partial solution, although essential for data discoverydoes not ultimately solve the underlying problem of outdated names persisting at the source, nor in other contexts that may not provide the safeguards GBIF does.

To date, neither data publishers nor consumers have a ready solution for persistently improving taxonomic content or tracking amendments, which continue to occur. Furthermore, the scope of the problem has not yet been properly quantified. Without that information, we cannot estimate the effort required to fix the legacy of problems at the source. Finally, although many efforts have been made to build informatics tools [35], it is not yet clear how well any particular automated solution works to fix taxon names (although see Vanden Berghe et al. [33] for some initial comparisons), nor is there a direct way to compare the effectiveness of those solutions.

A needed first step towards solving issues with taxon names is to develop standard taxon validation sets, which can be used to assess the type and scope of issues, and provide a human-vetted current name for a given taxon name input. In the present work, we develop a standard validation set for vertebrates based on content in the VertNet network [36,37]. VertNet works with a large community of biocollections data publishers to provision specimen and observation data records (18,255,043 occurrence records from 215 data resources containing 290 collections, shared from 91 data publishers globally as of 2 November 2015). All records published through VertNet are putatively harmonized to Darwin Core standards [12,13] and shared via installations of the Integrated Publishing Toolkit (IPT) [38]. We used vertebrate data exclusively for the current study as they are disproportionately digitized (both in percentage of total holdings and in terms of completeness, such as being georeferenced) compared to other groups. This makes these data more immediately ready for use, and therefore of priority for assessment of taxonomic quality. A carefully constructed validation data set can also serve to test hypotheses about what might drive taxonomic data issues. Here we provide such an assessment for vertebrates, focusing on how simple, potentially explanatory variables, such as year, region, higher-level taxonomic group, and institutional characteristics could account for different sorts of issues.

### Determining validity of taxon names

There are distinct types of issues that could arise with respect to taxon names. Chapman [39] provided a description of three main domains in which true taxon name errors can occur: identification errors, misspellings (in one or more taxon-related fields) and errors in format. The first type of error cannot easily be solved without validation against species range or distribution maps along with intervention of taxonomic experts, and is therefore outside the scope of this study. The second error domain, misspelling, can occur at many points in the process of communicating a name (initial labeling, transcription when digitizing, or transforming for data sharing). Misspellings can be defined as differences in a text string due to character insertions, deletions, substitutions or transpositions of what is otherwise correct [40].

The last of Chapman’s domains—issues with “format”—is a broad group of errors. The construction of a valid scientific name is regulated by specific nomenclatural codes, such as the International Code of Zoological Nomenclature [41], which establish the rules for name construction and usage at particular taxon ranks at a certain point in time. Errors in format can relate to violation of a code of nomenclature, and range from trivial, such as extra white spaces, to more difficult to solve, such as the representation of subspecific variants.

For the purposes of this study, we are also interested in a new error category not explicitly set apart by Chapman [39]: errors that arise from the misunderstanding or misapplication of the Darwin Core standard [12,13] field definitions or from the standard simply not being followed [42]. We refer to these errors as Darwin Core conceptual errors (not to be confounded with the term “taxonomic concept”). Darwin Core has become a de facto transmission format for digitally accessible biodiversity information [14]. For taxonomy in particular, Darwin Core has fields for scientific names and related information. Even though database curators,managers and biodiversity data aggregators are increasingly using the standard to publish their data, the content of the individual fields is not strictly regulated and is far from being homogeneous in practice. Common Darwin Core conceptual errors include using the scientificName field for binominals even when there is also an infraspecific epithet given, and putting identification qualifiers such as “sp.”, “cf.”, and “?” in one or more of the scientific name fields instead of in the Darwin Core field identificationQualifier.

While not strictly an error, a critical aspect of name validity is whether it is currently accepted by taxonomists in peer-reviewed literature. Taxonomists often synonymize names when they carry out taxonomic revisions, such as moving species from one genus to another or splitting groups so that name changes are required [43]. Biocollections data often contain invalid names that are not mistakes per se, but are junior synonyms. These should be updated to current valid names, especially because they can otherwise provide a distorted view of biodiversity by over- or under-representing taxon occurrences. We acknowledge that authors can have distinct taxonomic concepts [44,45], and that there are cases in which there are two or more alternative valid names for a given species. They represent the dynamic nature of our understanding of biodiversity.

In summary, our validation data set seeks to address four types of issues: misspellings, format errors, Darwin Core conceptual errors, and synonyms. These cover cases that can be automatically detected and corrected, cases that are easy to detect but less easy to correct (e.g., authorship mis-capitalization), and cases that cannot be corrected without careful vetting (e.g., valid names that do not exist in digital aggregated authorities, as often happens with subspecies or very recently described taxa). By investigating these types of issues in detail, we hope to find a better understanding of why they occur, the effort required to fix them, and the prospects for doing so automatically with sufficiently sophisticated tools.

## Methods

### Data acquisition

VertNet develops social and technical infrastructure to share vertebrate biodiversity data from natural history collections [36]. The network is comprised of data aggregated from self-published sources as well as sources published with the help of, and hosted by VertNet. Most vertebrate data hosted on the VertNet IPT (http://ipt.vertnet.org:8080/ipt/) [46] have first passed through customized data cleaning workflows, called "migrators", of the VertNet Darwin Core Migrator Toolkit [47], while self-hosted sources have not. The migrators map verbatim data from the source correctly to Darwin Core fields and provide standardized classifications to the level of genus by looking them up in a manually updated list of valid taxa [48], preserving the verbatim classification from the source in the Darwin Core field higherClassification. Thus, any study of the verbatim, unvalidated taxonomic content of data sets participating in VertNet must use the pre-migration data for those data sets that are hosted by VertNet, to ensure they are compatible in terms of data quality with datasets from self-hosted sources, which can be used as published.

**Fig 1:**
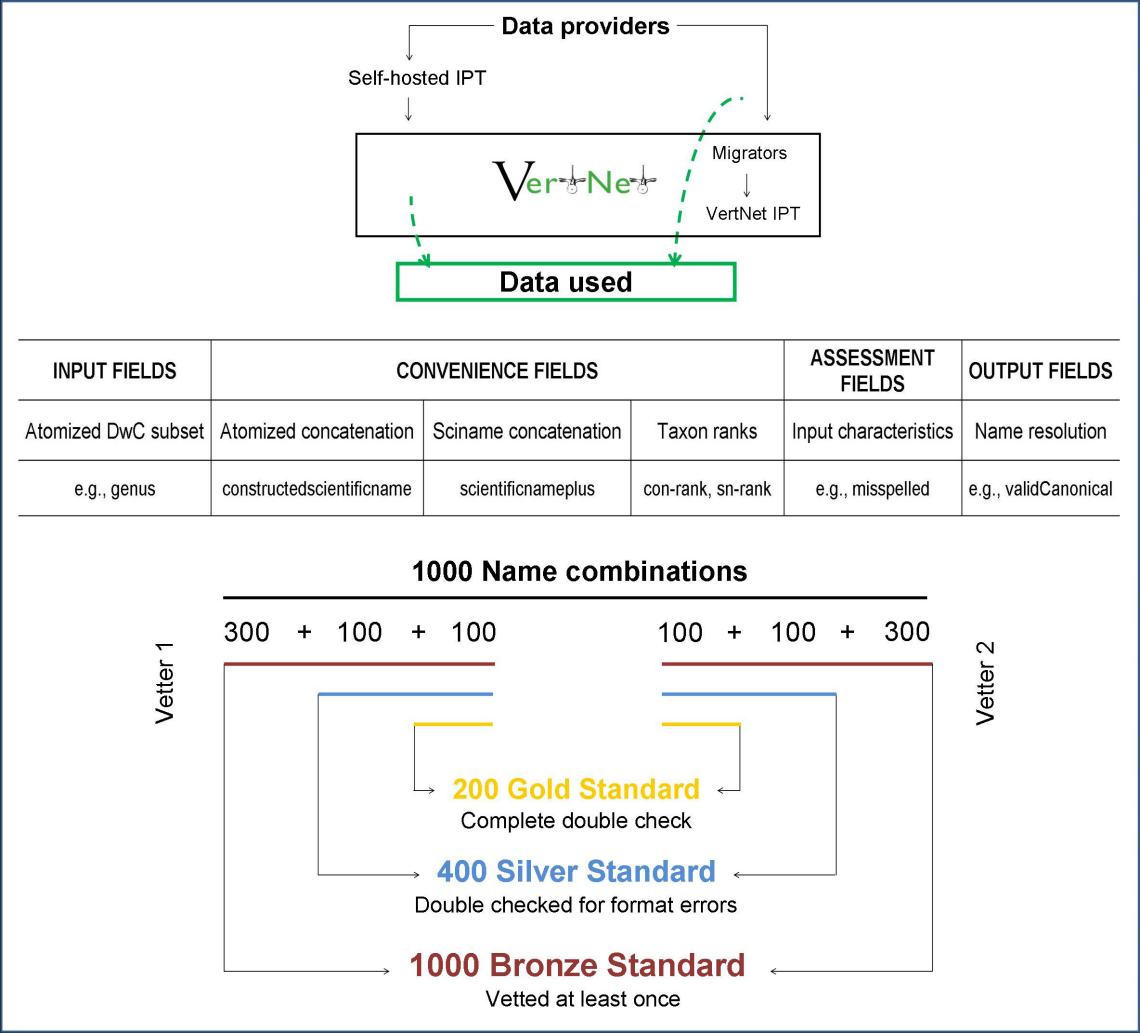
Data workflow to create the Gold Standard set of vertebrate names. Data from providers participating in VertNet were gathered directly from self-hosted data sets (top left), or, for data sets that undergo data cleaning through VertNet migrators prior to publication, as provided previous to migration (top right). The table (center) shows how data were organized in distinct fields for assessment. The composition and characteristics of the 1,000 name combinations in the data set (lower) distinguishes subsets resolved by each researcher (left and right), and cross-analysis performed by both researchers at different levels of certification (check for format errors only and complete double check). DwC: Darwin Core; Sciname: scientific name; **con-rank** and **sn-rank:** taxon ranks of **constructedscientificname** and **scientificnameplus** fields respectively.

In order to assemble migrated and non-migrated data sets (cited in S2 File), we queried the pre-migrator source data and the VertNet data store on 18 April 2015, following the protocols described in S1 File *Detailed VertNet names data acquisition*. The aggregation of data sets resulted in 522,163 distinct combinations of the verbatim information contained in the following Darwin Core fields: **scientificName**, **genus**, **subgenus**, **specificEpithet**, **infraspecificEpithet** and **scientificNameAuthorship** (Fig 1) (field names used in analysis are in bold to facilitate recognition in the text). These data were stored in a table to which we appended two fields, called **constructedscientificname** and **scientificnameplus**. The field **constructedscientificname**, designed to facilitate valid name resolution, was populated with the space-separated concatenation of the fields **genus**, **subgenus**, **specificEpithet**, **infraspecificEpithet**, and **scientificNameAuthorship**. In the cases where only the **infraspecificEpithet** field was populated with data, the **constructedscientificname** was left blank, as this condition was found to signal a particular interpretation of how to use Darwin Core fields and was accommodated separately with the scientific name (see below).

The field **scientificnameplus** was created to provide a corollary to the **constructedscientificname** based on the Darwin Core **scientificName** field. Inspection of the data set revealed that some verbatim names relied on a combination of **scientificName** and **infraspecificEpithet** to produce the full scientific name, apparently taking the **scientificName** field to be populated with nothing more than a binominal. Thus, for cases where **scientificName** and **infraspecificEpithet** were both populated and the **scientificName** did not contain an **infraspecificEpithet**, the field **scientificnameplus** was constructed from the space-separated concatenation of the two, otherwise it was populated only with the unchanged value in the **scientificName** field. From this table containing both verbatim data and the two concatenated fields, a random set of 1,000 distinct name combinations was taken (see S1 File *Detailed VertNet names data acquisition* for details). This sample data set contains an id field to identify distinct source name combination records.

### Organizing name issues

To organize the data, we defined four groups of fields, as described below (Fig 1, for detailed definitions of each field see S1 Table).

1. **Input:** verbatim information from the source (Darwin Core fields).
2. **Convenience:** concatenated subsets of the original fields that might be used by scientific name parsers and resolvers, plus taxon ranks for these: **scientificnameplus**, **dwcsn-rank** (taxon rank of the Darwin Core **scientificName** field), **sn-rank** (taxon rank of **scientificnameplus**), **constructedscientificname** and **con-rank** (taxon rank of **constructedscientificname**.
3. **Assessment:** errors in detectable characteristics of the name record (e.g., mis-capitalizations, extra spaces, misspellings, etc.). These include fields related to the **scientificnameplus** and to the **constructedscientificname**, as those characteristics were tracked for both concatenations. Also included are the fields **hasIssue**, **isSynonym**, **hasMisspelling**, **hasConceptualError** and **hasFormatError**, where we summarize the occurrence of different types of issues for further analysis (see below).
4. **Output:** results of the resolution of the scientific names and the reference to the consulted sources. They include: **validCanonical**, **validSource**, **sourceURL**, **sourceDate** and **comments**.

### Assembly and initial validation of the data

We used regular expression matching to check for simple errors that can be automatically detected and recorded (e.g., unexpected extra characters or spaces and other formatting errors). These were catalogued in the appropriate Assessment fields and would later be checked and amended if necessary during manual vetting. After this first pass, the 1,000 name combinations were divided randomly into two distinct sets of 500 names each, with one set assigned to each of two authors for full vetting. Each vetter’s goal was to determine the validity of the given names, to track issues found and to provide a valid canonical name and source from which it was obtained.

For validation we consulted several authoritative online sources (see S3 Table) and proceeded as follows: 1) in most cases we chose to list specialized sources over general aggregators (e.g., The Reptile Database over ITIS or CoL) 2) when sources differed in accepted valid canonical name, we favored the result with the highest representation in distinct sources or the source with the mot recent revision date; 3) rather than give an exhaustive accounting, we listed one or two resources that contained the valid name for citation purposes.

In order to test the vetting process for consistency, the data manager independently shared 50 name combinations with both vetters to resolve fully. This exercise yielded differences for some records between the results from the two vetters and served to harmonize how issues related to inclusion of scientific name authorship within the **constructedscientificname** field should be treated. In some cases, *only* **scientificNameAuthorship** was found in the **constructedscientificname** field. In these cases we did not consider the content of this field to be invalid if the authorship information was well-formed and accurate (e.g., correct author names and date). If **constructedscientificname** contained some kind of error (e.g., incorrect dates or mis-capitalizations), we did consider that content to be invalid.

After this initial test, which showed ~85% consistency between vetters, a second subset of 24 name combinations, not used in the first subset, were shared and resolved by both vetters using the refined methods from the first pass to determine if results were reasonably in accord. This yielded >95% overlap, with small differences in synonyms reported and further discussed below.

### Assembling the full validation data set

Once the vetting process was tested, subsets of 100 names were given to each vetter, with each subset containing 20% of names in common between the two. When a subset was processed, the names in common were compared to check for consistency in the use of the methodology. Thus, when all subsets were completed, 200 name combinations were fully resolved by both vetters. These 200 names were carefully re-checked by both vetters to determine the causes of any inconsistencies and arrive at a consensus. In the vast majority of cases where there was inconsistency, there had been a mistake by one vetter or the other that was easily fixed. In a small number of cases, issues with competing, presumed valid scientific names were uncovered, as reported in the Results section.

As an additional check of consistency, two subsets of 100 records, each taken from the once-vetted set of 400 from each vetter, were shared with the other vetter for review. This review covered assessments of validity, errors in the input, and assigned ranks of the output, but did not include consulting the documented sources to assess the assignment of the final valid taxon name (field: **validCanonical**). This pass revealed only two minor inconsistencies among the 200 reviewed names, which were amended. Finally, each vetter reviewed the remaining 300 of his/her own name combinations for issues, which revealed only about ten additional minor inconsistencies, also amended for the construction of the final data set.

With the results of the two independent assessments and the cross-checks, we categorized names that had passed through varying levels of review. The 200 gold standard name combinations were vetted by at least two authors for all issues, including full checks for synonyms and misspellings. The 400 silver standard names include the 200 ones that were fully checked plus 200 that were checked for common format problems by both vetters, but were not checked for synonymy or misspellings. Finally, the whole set of 1,000 name combinations constitute the bronze standard set, as they were all carefully examined by at least one researcher. For the published final data set we added a field (**checked**) to denote which records fall into each category.

### Assembling matching occurrence data

Once vetting was complete, the original taxon name combinations were linked back to collections records in VertNet. The link was made by creating an identifying taxon name key value from the concatenation of all six input fields. The matching occurrence records were then found using a database inner join between the taxon name key fields in the names table and in the occurrence table, and were extracted and aggregated in a new table, MatchingOccurrences. This process allowed us to count how many original VertNet biocollections records corresponded with each name combination, as well as to gather data about each of those records (e.g., year collected, country of collection, institution, etc.) All this information was used, as described below, to test variables that might affect taxon data quality.

### Assembling summary data

The validation data set provides information about prevalence of distinct types of taxonomic name problems commonly found in aggregated biocollections data grouped in our four categories: misspellings, format errors, Darwin Core conceptual errors, and synonymy. All detailed issues and their mappings to these broader categories are shown in S1 Table. As an example, we group **con-authcap**, which captures whether the authorship was correctly capitalized in accordance with the ICZN, among the format issues. In contrast, we group **con-autherr**, which denotes whether an author name shows up in the **specificEpithet** or **infraspecificEpithet** fields instead of being properly placed in the **scientificNameAuthorship** field, among the Darwin Core conceptual errors. We counted the number of name combinations in which a given type of issue arose in the validation set (S2 Table) as well as how many name combinations exhibited issues as grouped within the four larger categories.

### Testing factors that explain taxonomic issues

We used logistic regression generalized linear models to determine how distinct predictors drive the prevalence of issues in taxonomic name combinations. In particular, we used the following independent variables: value of the field **basisOfRecord**, geographic region, clade, number of digitized vertebrate records shared by an institution, and **year** of collection. For these analyses we used information from all records matching the name combinations, obtained as described above in **Assembling matching occurrence data**. To standardize the content of fields used in our analysis, the following actions were taken: 1) **basisOfRecord** was standardized to the recommended Darwin Core controlled vocabulary, and from it, only FossilSpecimen and PreservedSpecimen records were retained; 2) geographic and date fields were first standardized based on the criteria of the VertNet Darwin Core Migrator Toolkit [47], and then the region was extracted for both terrestrial and marine locations; regions considered include: Africa, Asia, Australasia, Europe, North America, Oceania and South America; we did not consider the records from Antarctica, as they were few and strongly restricted by clade; 3) clade was set to one of five categories: Amphibia, Aves, Mammalia, Reptilia and “Fishes”, the latter grouping all records belonging to the clades Actinopterygii, Cephalaspidomorphi, Conodonta, Elasmobranchii, Holocephali, Myxini, Placodermi and Sarcopterygii; 4) any record for which geographic region or year could not be determined was not used and; 5) for the synonymy cases, we did not use name combinations for which we could not unequivocally determine if the given name was a synonym or not (i.e., cases in which the given name was not found in authoritative sources).

First, we investigated how the distinct predictors affect the overall prevalence of issues in the name combinations, captured in the **hasIssue** field (i.e., name combination has at least one issue). We constructed models using R’s glm function [49], considering all possible combinations of two-way interactions between predictors. In order to select a best model for the one that included two-way interactions, we compared the ordered AIC values [50] of all the possible models, including 0–10 (maximum) 2-way interactions, using the R MuMIn package [51]. Nagelkerke’s and McFadden’s *R*^*2*^ value were calculated using the R BaylorEdPsych package [52]. Effects for the chosen model were plotted using the R effects package [53].

In order to determine how each distinct type of issue affects the validity of name combinations, we fit main effects GLM linear regression models for each issue separately, selecting the best model according to best AIC criterion as before, using MuMIn. For these models, we only considered main effects of the variables, as the interactions among them (as seen from the models for the general “Has issue” case) complicates comparative conclusions. The general trends derived from these models were qualitatively compared to those derived from the main effects model for “Has issue”.

We expected significant interactions among the distinct predictors tested, in line with the hypotheses on how each variable affects the prevalence of issues in the taxonomic names, given below. BASIS OF RECORD: We expect that fossil specimen records will show higher prevalence of issues than preserved specimens records, given the high rates of synonymy that have been observed for certain fossil groups [54]. CLADE: We expect higher-level clade (i.e., taxon rank of class) to have a significant effect on the response variable, and particularly on the occurrence of synonymy, since some taxa may have faster rates of redescription than others [55]. REGION: Some regions are more poorly known in terms of biodiversity, and in such cases lack of resources may limit name curation and increase the prevalence of issues. We therefore expect North American and European records to have the lowest issue rates. YEAR: We hypothesize that names associated with older records are prone to have more errors for two reasons: 1) data taken from older labels and ledgers are more likely to have an out-of-date name, all else being equal, simply because the chance of names changes in intervening time period is higher, assuming that most labels are not re-identified in the interim; 2) it is more difficult to read older labels, which likely leads to higher rates of misspelling.

It is possible that record invalidity has less to do with an individual aspect of the record, and more with the digitization methods and curation practices of institutions and collections from which the data set is drawn. We therefore also tested DIGITIZED COLLECTION SIZE of an institution, and considered two contrasting hypotheses. Institutions with larger digitized collections may have proportionally less staff time to devote per specimen at all stages of digitization and revision. Conversely, those institutions with more digitized records may have greater overall expertise in collections digital data management, thus reducing the prevalence of certain types of issues.

## Results

### Characterizing the data set

We examined 1,000 vertebrate name combinations associated with a total of 27,113 species occurrence records aggregated in VertNet (Table 1). These name combinations belong to 12 distinct classes (S2 Table), of which Aves and Actinopterygii were the most numerous (359 and 327 name combinations respectively). In the names resolution process, the two researchers consulted 73 combinations of 50 distinct authoritative sources (S3 Table). Of the 1,000 input names combinations, we were able to provide **validCanonical** names for 967 (96.7%) of them, corresponding to 26,995 occurrences (99.6%) from all 71 contributing institutions (Table 1). The 33 name combinations for which we could not provide a **validCanonical** name corresponded to 118 occurrence records from 19 institutions. It is possible that further efforts by experts and investigation of the original specimens could resolve more of these 33 currently unresolved names.

**Table 1:** Summary information on the 1,000 name combinations that form the basis of the validation set.

	Name combinations	Matching Occurrences	Institutions	Data sets
Total	1,000 (100%)	27,113 (100%)	71	142
Valid name found	967 (96.7%)	26,995 (99.6%)	71	142
Valid name not found	33 (3.3%)	118 (0.4%)	19	23
Has error (at least one)	341 (34.1%)	3,207 (11.8%)	50	78
Has issue (at least one)	532 (53.2%)	6,867 (25.3%)	61	105
Misspelling	129 (12.9%)	775 (2.9%)	37	52
Format error	89 (8.9%)	1,061 (3.9%)	16	19
Conceptual error	137 (13.7%)	1,458 (5.4%)	36	51
Synonym	270 (27%)	4,147 (15.3%)	54	92
Indeterminate	41 (4.1%)	392 (1.4%)	23	29

The number of name combinations that were initially valid, or could be validated, are shown, along with detailed prevalence of problems, such as the number that were synonyms, or had format errors or misspellings. We also present how those names are related to matching occurrences, and their associated data sets and institutions. Except for the three first rows, numbers do not include results for the 9 name combinations for which researchers could not arrive at a unique resolution. Note that “Has error” differs from “Has issue” in that the latter includes the occurrence of synonymy (which is not strictly an error).

The comparison of the resolution of the 200 name combinations that were independently assessed by two researchers showed concordance to a single valid canonical name in 191 cases. For the remaining 9 cases, no agreement was reached for a single valid canonical name, as it was considered that either of the results could be correct depending on the authoritative source consulted. Based on this result, we decided not to consider these 9 name combinations when performing statistical analysis and modelling, but we do include the “double resolution” in the general data set available for the public to use. For this reason, the reference data set contains 1,009 name records, with 9 records being duplicated to show the independent resolutions. We should note that we expect many name combinations in the current data set that have a single current resolution may prove to have alternate opinions in different taxonomic sources. We also note that taxonomic opinions on all the names in the data set are subject to change as a result of further taxonomic research. Although we cannot guarantee completeness in the reference set, we did select from the most recent resources, often checking more than one for consensus on current usages. Our decision to seek a consensus approach reflects our view that the downstream user’s dominant use case is to have a single name, where possible, associated with records.

**Fig 2:**
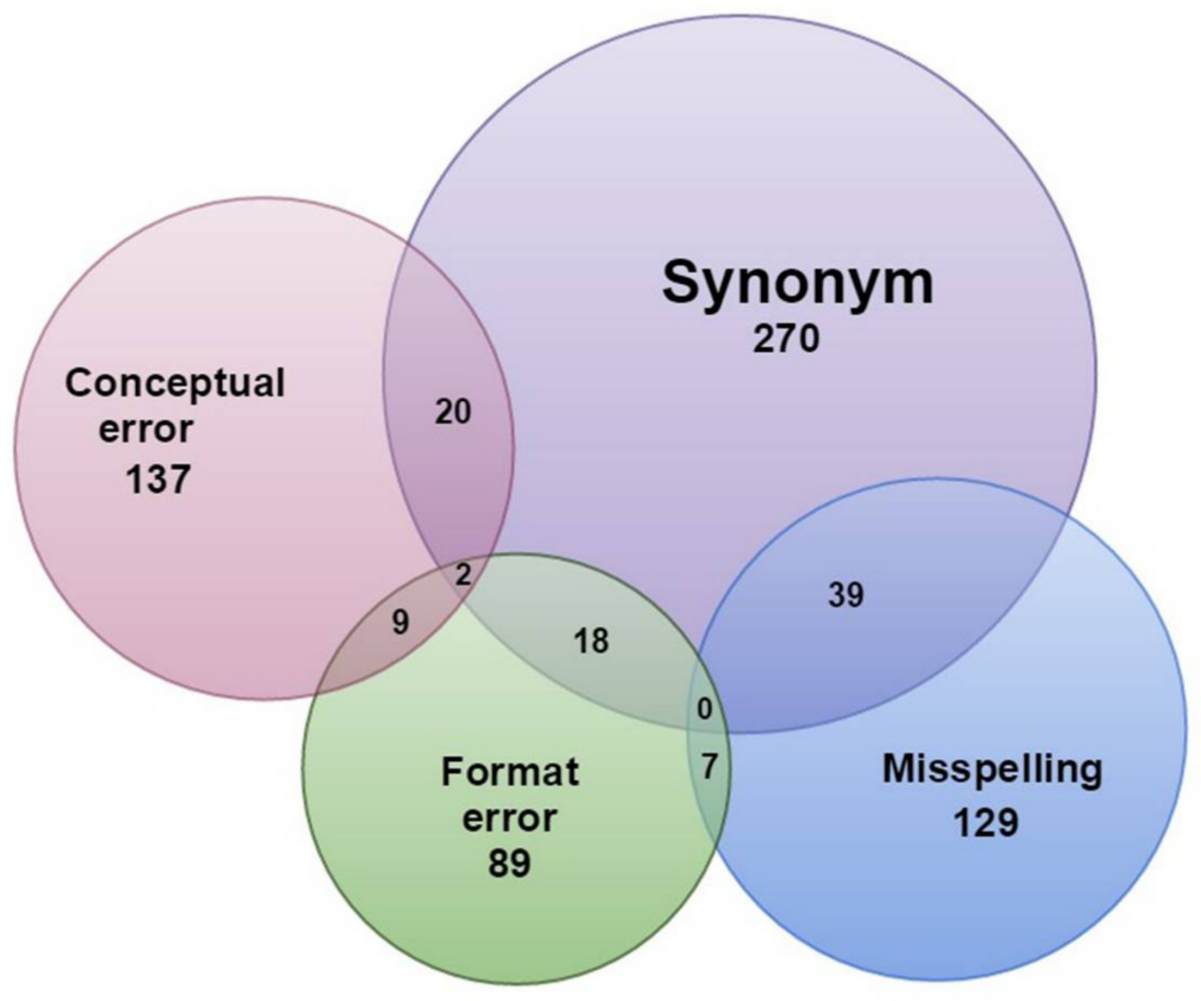
Summary of issues encountered in the name combinations that form the basis of the validation set. Numbers indicate name combinations that showed one or more types of issues. Total number of name combinations assessed for issues = 991, total number of those name combinations with issues = 532, total number of those name combinations with errors (misspelling, conceptual or format error) = 341.

In order to characterize the data and to determine the rate at which different kinds of errors occur, we used the distinct fields described in S1 Table. Errors (a subset of issues) were found in 341 of the 1,000 name combinations (11.8% of matching occurrence records, 70.4% of contributing institutions, Table 1). There were a total of 488 distinct issues in these 341 name combinations, detailed in Fig 2 showing overlaps of issue types. The detailed numbers for each case can be found in S2 Table. Table 1 shows a summary of issues in various categories. The most common issue was synonymy, of which we found 270 definitive cases (Table 1, Fig 2). In 41 cases we could not determine whether the name was a synonym or not. We found 129 cases of misspelling. Synonomy and misspelling do not always cleanly separate, especially in cases of improper latinized gender endings. The incorrect use of Darwin Core, which we characterized as conceptual errors, was the second most common type of issue encountered (137 cases). Finally, format errors were encountered in 89 cases.

### Testing factors that explain taxonomic invalidity

We constructed generalized linear models for the prevalence of at least one issue (“Has issue”) and for each type of issue separately, using basisOfRecord, Geographic Region, Clade, Institutional digital accessible vertebrate records count, and Year of collection as explanatory variables. The model for “Has issue” considering all 2-way interactions between the variables was better than the model that only considered main effects (AIC = 17,342 and 21,510 respectively), or a more limited set of 2-way interactions, and hence was the model we chose. The *R*^*2*^ values for this model were: McFadden’s *R*^*2*^ = 0.257 and Nagelkerke’s *R*^*2*^ = 0.378. Plots for the interactions between the predictors for this models are shown in S1, S2 and S3 Figs.

Results from this model (S1, S2 and S3 Figs) show that:

1. basisOfRecord: fossils are more prone to issues than non-fossil preserved specimens.
2. Geographic region: South America is more prone to issues, particularly for older records.
3. Clade: Amphibia is more prone to issues than any other clade, “Fishes” are less prone to issues.
4. Institutional digital accessible records count: institutions with larger records count are less prone to issues.
5. Year of collection: older records are more prone to issues, particularly in smaller institutions and for the clades Amphibia, Reptilia and Aves; year affects fossils and non-fossil preserved specimens equally.

**Fig 3:**
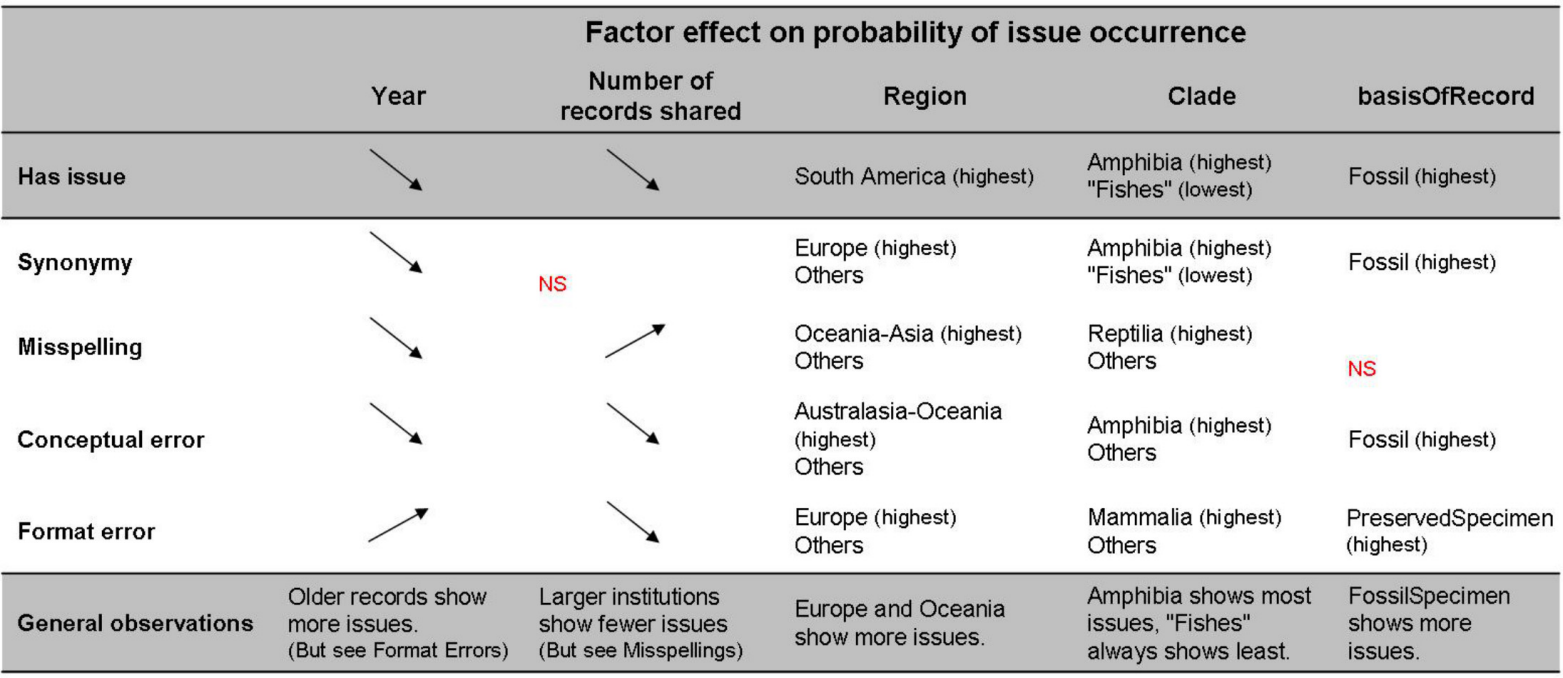
Main effects of the predictors on the probability of occurrence of issues on the taxonomic name combinations. Factors: basisOfRecord, Geographic region, Clade, Number of records shared by Institution and Year. Issues: Synonymy, Misspellings, Conceptual errors, Format errors. “Has issue” denotes the presence of at least one of the cited issues. Effects calculated through logit GLM, with binomial response. For definition of the “Fishes” clade, see text. NS: statistically not significant.

In order to compare the prevalence of distinct types of issues, we explored the main effects of the predictors for each issue. For all four types of issues the best models, as found from comparison of AIC values (not shown), were those which included all predictors. The prevalence of each distinct issue (Fig 3) showed similar patterns to those found for “Has issue” for most predictors: fossils are more prone to all types of issues; Amphibia show the highest probability of most issues, while the probability is low for “Fishes” across all types of issues; institutions with a larger number of digitized records have a lower probability of issues, with the exception of misspellings; older records have higher probability of issues, except for format errors. For geographic region, however, analysing each issue separately showed that North American records appear to have the lowest error rates.

## Discussion

We present a human-vetted data set of 1,000 verbatim vertebrate scientific name combinations and their corresponding valid canonical names, along with an assessment of the prevalence and potential explanatory variables for various error types. Another approach taken in recent work by Vanden Berghe et al. [33] was to utilize their own taxonomic authority files (TAF) as a validation set. While the TAF approach scales well, a human-vetted approach provides stronger assessment of name validity across sources, and provides a much more detailed, granular vetting of the type of problems encountered. It also provides another sort of benchmark—the effort required to manually clean names, further discussed below. Overall, our results show that, although 97% of the name combinations could be resolved, over 53% of those names and over 25% of the associated species occurrence records exhibit at least one issue (errors and/or synonymy). Our work shows that, while digital information for taxonomic name resolution is accessible, there is urgency for vertebrate taxonomy name cleaning.

### Characterizing problems in digitized scientific names

We have shown that three of the strongest predictors influencing prevalence of issues with digitized names associated with vertebrate biocollections are year, clade and record-type. The effect of clade on the overall occurrence of issues showed Amphibia have the highest probability of issues, and “Fishes” the lowest. We believe this trend is mainly driven by synonymy prevalence (see below), as avian, and especially amphibian revision rates are known to be very high [55]. This could explain why there is a threefold difference between the probability of amphibian digitized records having an issue compared to fish (~0.45 probability of error compared to 0.15, see S1, S2 and S3 Figs).

Collection year is also a relatively strong predictor. Specimens collected less recently have a higher probability of having name issues than more recent ones. This result for year holds for all issue categories except format errors, which is treated more in detail below. Our analysis also shows that fossil specimens are more issue prone than preserved specimens. High probability of synonymy in fossils might be attributable to high revision rates, as has been seen for fossil mammals [54], while the high probability of Darwin Core conceptual errors might be driven by data sharing practices using Darwin Core being a relatively new activity in the paleontological community compared to its use in other disciplines.

While there are significant differences among regions where a specimen was collected, we note that a simple hypothesis, that records from regions where the biodiversity is more poorly known have more issues, is not strongly supported. Since VertNet records are drawn mostly from US institutions (78.9% in our data set), it is perhaps unsurprising that records from North America have lower error rates (e.g., misspellings and format errors) than records from other regions, which might suggest that more curatorial emphasis is given to domestic records than to foreign ones, or that domestic ones are easier to curate, given better availability of local or regional resources. Analogous studies of collections from institutions in other regions of the world would shed further light on the effect of region on the prevalence of issues.

A final predictor is the number of digitally accessible occurrence records shared by the institution from which the records came. This variable does explain probability of issues, with those institutions with larger record volume having a lower prevalence of issues. We conclude that for larger institutions, the practicalities of handling large numbers of specimens and data might be overcome by having greater overall resources for collection management and biodiversity informatics.

### From documenting rates and drivers to long-term solutions

The high prevalence of synonymy is perhaps not too surprising, since this type of issue is the most difficult one to solve. Unlike other issues, synonymy is neither an error, nor static. Managing the problem is highly dependent on the current knowledge of taxa, the rate at which different groups are revised, and the availability of resources to track nomenclatural changes. This issue is affected by the preference any particular curator might have for one source or another, and assigning a valid name can also be challenging in cases where there is still taxonomic disagreement (as shown, for example, for birds) [21]. Further refinement of names and the taxon concepts they represent in nature is ongoing, and at similar or higher rates than ever before, even more so in the best studied groups [54]. We conclude that in order to keep issues with synonymy at a minimum, it is indispensable to make periodic checks of taxon names. Such checks should include the date on which the update was performed, sources consulted, and the nature of the change (taxonomic revision versus re-identification). The approach to updating label names to currently valid ones has been haphazard in curatorial practice [56], but it can become more formalized and potentially more automated as data become more aggregated, resources become easier to access programmatically, and collective standards emerge in the community.

The second most common type of issue encountered was failure to follow Darwin Core standards (conceptual errors) when populating the fields that are publicly shared. This result is surprising when considering that Darwin Core, a ratified standard since 2009, provides definitions of all terms and clear guidelines on what the content of each field should be. Our results show that the type of information published in a given Darwin Core field can vary. The high prevalence of these conceptual errors begs for solutions from both data publishers and standard-makers in two ways: 1) standards are not being followed, and that can result in data not being discoverable and available for use even when it is being publicly shared, 2) standards might not be clear or emphatic enough with respect to best practices. There are a couple of ways in which Darwin Core conceptual errors could be mitigated. First, the Darwin Core standard materials should provide a complete set of clear examples of what to do and what not to do to supplement the definitions of terms that already exist, since the definitions alone apparently do not go far enough to achieve the goal of well-mapped data. Second, best practices should be promoted by data managers and data consumers alike, using the above-mentioned examples through data quality tests and feedback.

One challenge is that not all specimen data management systems provide the same conceptual distinctions as Darwin Core. For example, although Darwin Core distinguishes fields for names from those used in taxonomic determinations of specimens (e.g., terms in the Taxon and Identification classes), a common problem we encountered is the inclusion of identification qualifiers (sp., ssp., cf.,?) along with the names in Taxon fields. Extracting and correctly mapping these qualifiers from the name fields in the original data into Darwin Core would require special parsing, and this is one technical step more difficult than simply mapping a source data field directly to a Darwin Core equivalent. One practical solution is for data managers to adopt Darwin Core fields in their source databases in places that make sense for their daily practices, but if there is no compelling reason for them to make such distinctions in-house, the problem will necessarily travel downstream. Another solution to this problem is more fully attained in VertNet by using the VertNet Darwin Core Data Migrator Toolkit described earlier [47], which ensures that the Darwin Core conceptual errors are corrected, if not at the source, at least at the point of public access, the published data resource.

Format errors included incorrect capitalizations, addition of extra white spaces, and abbreviations. This type of error does not originate in curatorial content parsing nor does it propagate from original labels or ledgers. Although they follow the general trends for most predictors analyzed, format errors occur with higher probability in more recent records. However, these errors are readily solvable by automatic processes, at least when not combined with other types of errors.

The final class of errors we considered is misspellings, which we found in 13% of the name combinations. As expected, records with older dates of collection are more likely to be misspelled. Contrary to other issue trends, institutions with larger numbers of digitized records have proportionally more misspellings. It may simply be that larger collections are more likely to have proportionally more old records or less care can be taken per record especially when laboriously digitizing content (as opposed to digitally curating that content, which can be done more in bulk). While 13% of name combinations are misspelled, the proportion of digitized collections records bearing those misspelling is smaller, representing 2.9% of the total number of records. This rate may not seem particularly high, but when extrapolating to the whole of VertNet, it yields ~500K misspelled records, most of which can likely be correctly determined with smart fuzzy matching algorithms such as TaxaMatch [57]. A critical future challenge is to develop tools that can detect synonymy and misspelling issues simultaneously, since in many records we found misspelled junior synonyms requiring approaches that would involve multiple stages of cleaning.

### Understanding the effort required to improve taxon names

Although we did not measure the exact time taken to clean each of the 1,000 name records by hand, the two authors who performed the validation step estimate it took each person 4 hours of concerted work to complete a set of 100 records. The resolution of a typical name record (which includes two name combinations, **constructedscientificname** and **scientificnameplus**) took approximately 2 minutes to complete, with the more challenging records taking significantly longer. Manually documenting errors and validating all the name combinations in VertNet, assuming that our sample is representative, would thus take 20,887 person-hours (or 10.4 years for an average work schedule) just for a single-person validation (i.e., equivalent to the bronze standard). Our effort calculations are likely an overestimate of the actual time to minimally assess taxon names, since rarely is the goal to provide such a thorough assessment of problems. However, if the goal is to to create the cleanest taxonomic data possible for providers and consumers, our effort calculations only provide one part of the story, since effort is also needed to coordinate with the provider network in order to improve data both at the source and across VertNet. This effort estimation presents two main biases. First, it is based on vertebrate data, which hold a relatively small pool of names and benefits from the availability of relatively robust digital authority sources for validation, in comparison with other biological collections (i.e., entomology). Secondly, it only applies to data that are already in a digital format. While corrections on data in digital format can be simultaneously applied to sets of records (i.e., corrections in a name can be applied to all matching occurrences at once), correction on the original labels and ledgers may be much more time consuming, as they need to be located and changed on individual specimens in collections of distinct institutions. The number of records shared by institutions and the number of people dedicated to curation play a key role in the implementation of label curation. It has to be noted, though, that not all types of issues are of real interest at the collection level, for example, format and conceptual errors lack meaning for specimen labels.

### Building better data cleaning approaches

Cleaning scientific names associated with labels can be approached either from an holistic perspective, trying to solve all issues for a given name, or from a more stepwise, cumulative perspective, trying to solve one kind of issue at a time in a procedural series. Our results show that the prevalence of problems in taxonomic names is distinct for each type of issue, and can be affected differently by distinct factors and their interactions. Thus, tools are likely required that can both recognize issues and understand record contents in full, e.g., higher level taxonomy along with all of the input fields used in this study.

A critical recommendation from this work is that tools should not simply focus on synonymy or even just synonymy and misspellings together. Instead, tools should be developed that can detect and remedy all combinations of the errors we have reported above, and be able to parse them and report issues types back to data providers and end users, along with recommendations for improving the data. These tools should become an integral part of the ecosystem of data publishing for biodiversity records of all types, and help enforce the kind of community practices that minimize format and Darwin Core conceptual errors. As a general recommendation for building and using data parsers and resolvers, we emphasize that the resolution of taxonomic names should not be limited to the analysis of individual Darwin Core fields. As seen in our study, full names resolution often requires gathering information from all of the taxon fields (e.g., **scientificName** and **infraspecificEpithet** when the infraspecific epithet is not included in the **scientificName** field). Considering and cross-referencing as many fields as possible would render better chances of obtaining a correct valid name for a given name combination.

## Conclusion

If not corrected, errors and out-of-date names in taxonomy data can be easily propagated from databases through scientific research studies, impacting the results of ecological and environmental studies, which in turn affect conservation policies [58]. The problems we report here are focused on vertebrate biocollections, but they also plague any sort of biodiversity data set containing taxon names across all named species, ranging as widely as from species inventories data to taxon names associated with genetic data. We therefore encourage communities to develop validation data sets for other taxa and data types, with similar care, transparency and focus on categorizing types of issues. Biodiversity informatics tools for provisioning biocollections records have matured enough to bring this critical legacy into a Big Data framework, but the problem we address is very general, requiring general solutions. While this small contribution of a carefully constructed validation data set for vertebrate names is only an incremental step forward, we hope it will help catalyze the community to both further assess drivers of these issues and continue developing smart approaches to data cleaning. Such approaches should, at best, generate outputs as close as possible to what a knowledgeable human with access to digital reference tools can produce. We think there is ample reason to believe this is achievable, and we encourage tool developers to prove this supposition using our validation data set as a key part of their testing suites.

## Supporting Information

S1 Fig Has issue. Effect of the interaction of the variables basisOfRecord, Geographic region (shown as “continent”), Clade (shown as “cladedf”), Volume of records shared by Institution (shown as “RecordsCountPerInst”) and year on the probability of occurrence of at least one of the following issues: Synonymy, Misspelling, Conceptual error, Format Error. Effects calculated through logit GLM, with binomial response using the R package “effects”. **A**: basisOfRecord:year; **B**: basisOfRecord:Clade; **C**: basisOfRecord:Volume of records shared; **D**: basisOfRecord:Geographic region.
(TIF)

S2 Fig Has issue. Effect of the interaction of the variables basisOfRecord, Geographic region (shown as “continent”), Clade (shown as “cladedf”), Volume of records shared by Institution (shown as “RecordsCountPerInst”) and year on the probability of occurrence of at least one of the following issues: Synonymy, Misspelling, Conceptual error, Format Error. Effects calculated through logit GLM, with binomial response using the R package “effects”. **A**: Year:Clade; **B**: Year:Volume of records shared; **C**: Year:Geographic region; **D**: Clade:Volume of records shared.
(TIF)

S3 Fig Has issue. Effect of the interaction of the variables basisOfRecord, Geographic region (shown as “continent”), Clade (shown as “cladedf”), Volume of records shared by Institution (shown as “RecordsCountPerInst”) and year on the probability of occurrence of at least one of the following issues: Synonymy, Misspelling, Conceptual error, Format Error. Effects calculated through logit GLM, with binomial response using the R package “effects”. **A**: Clade:Geographic region; **B**: Volume of records shared:Geographic region.
(TIF)

S1 File Detailed VertNet Names Data Acquisition (DOC)

S2 File Supplementary References. (DOC)

S1 Table Fields used to construct the Reference Data Set for Vertebrate Taxon Name Resolution. Fields are grouped into the four categories used in the study: Input, Convenience, Assessment and Output. Note that, among the assessment fields, **sn-inf-missing** only applies to **scientificnameplus**, and **con-autherror**, **con-rnk** and **con-sgerror** only apply to **constructedscientificname** since Darwin Core Standard does not by definition restrict the taxon rank to which **scientificName** can resolve to. In observations, we show to which type of issue we assigned each relevant assessment field. For evaluation of synonymy, comparisons were made between the contents of the Input and Convenience fields with the **validCanonical** name, and results were put into **isSynonym** field. For definitions of conceptual and format errors, see text. **con-**: referring to the field **constructedscientificname**, **sn-**: referring to the field **scientificnameplus**; DwC: Darwin Core Standard.
(DOC)

S2 Table Detailed assessment of the 1000 name combinations. Number of name combinations for which a field or characteristic of the name combination matched a given condition. Numbers outside of parentheses are for the 991 name combinations for which there was no disagreement between the assessment of the two researchers. Numbers in parentheses are the counts for the results that had multiple opinions on valid names. For definitions of fields, see S1 Table. * Classes included in “Fishes” clade for analysis of drivers of issues.
(DOC)

S3 Table Taxonomic Name Sources Consulted. Number of name combinations for which distinct taxonomic name sources provided the name given in validCanonical. We do not include 30 sources that were only consulted once. Some name combinations were checked utilizing multiple sources, and in some cases secondary sources used for confirmation were not always captured by the vetter.
(DOC)

## References

[1] Graham CH, Ferrier S, Huettman F, Moritz C, Peterson AT. New developments in museum-based informatics and applications in biodiversity analysis. Trends Ecol Evol 2004; 19(9):497–503. 16701313 10.1016/j.tree.2004.07.006

[2] Jetz W, McPherson JM, Guralnick RP. Integrating biodiversity distribution knowledge: toward a global map of life. Trends Ecol Evol 2012; 27(3):151–159. 10.1016/j.tree.2011.09.007 22019413

[3] Hill AW, Otegui J, Ariño AH, Guralnick RP. Position Paper on Future Directions and Recommendations for Enhancing Fitness-for-Use Across the GBIF Network, version 1.0. Copenhagen: Global Biodiversity Information Facility, 2010; 25 pp. ISBN:87-92020-11-9.

[4] Boakes EH, McGowan PJK, Fuller RA, Chang-qing D, Clark NE, O’Connor K, et al Distorted views of biodiversity: spatial and temporal bias in species occurrence data. PLoS Biol 2010; 8(6): e1000385 10.1371/journal.pbio.1000385 20532234 PMC2879389

[5] Maldonado C, Molina CI, Zizka A, Persson C, Taylor CM, Albán J, et al Estimating species diversity and distribution in the era of Big Data: to what extent can we trust public databases? Global Ecol Biogeogr 2015; 24:973–984. 10.1111/geb.12326 PMC5012125 27656106

[6] Guralnick RP, Hill AW, Lane M. Towards a collaborative, global infrastructure for biodiversity assessment. Ecol Lett 2007; 10: 663–672. 17594421 10.1111/j.1461-0248.2007.01063.x PMC2040220

[7] Page RDM. Biodiversity informatics: the challenge of linking data and the role of shared identifiers. Brief Bioinform 2008; 9(5):345–354. 10.1093/bib/bbn022 18445641

[8] Hjarding A, Tolley KA, Burgess ND. Red List assessments of East African chameleons: a case study of why we need experts. Oryx 2014. Cambridge University Press (CUP).

[9] Patterson DJ, Cooper J, Kirk PM, Pyle RL, Remsen DP. Names are key to the big new biology. Trends Ecol Evol 2010; 25(12):686–691. 10.1016/j.tree.2010.09.004 20961649

[10] Kennedy J, Hyam R, Kukla R, Paterson T. Standard data model representation for taxonomic information. OMICS 2006; 10(2): 220–230. 16901230 10.1089/omi.2006.10.220

[11] Deck J, Guralnick R, Walls R, Blum S, Haendel M, Matsunaga A, et al Meeting Report: Identifying practical applications of ontologies for biodiversity informatics. Standards in Genomics 2015; 10:25 [10.1186/s40793-015-0014-0].

[12] Wieczorek J, Döring M, De Giovanni R, Robertson T, Vieglais D. Darwin Core. [Internet]. 2009. Accessed 2015 Aug 17. Available: http://www.tdwg.org/standards/450/.

[13] Wieczorek J, Bloom D, Guralnick R, Blum S, Döring M, Giovanni R, et al Darwin Core: An Evolving Community-Developed Biodiversity Data Standard. PLoS ONE 2012; 7(1): e29715 10.1371/journal.pone.0029715 22238640 PMC3253084

[14] Meyer C, Kreft H, Guralnick RP, Jetz W. In Press. Global priorities for an effective information basis of biodiversity distributions. PeerJ 2015; PrePrints 3:e1057. 26348291 10.1038/ncomms9221 PMC4569846

[15] Global Biodiversity Information Facility (GBIF). [Internet]. 2015. Accessed 2015 Jul 2. Available: www.gbif.org.

[16] Boyle BL, Hopkins N, Lu Z, Raygoza Garay JA, Mozzherein D, Rees T, et al The taxonomic name resolution service: an online tool for automated standarization of plant names. BMC Bioinformatics 2013; 14:16 10.1186/1471-2105-14-16 23324024 PMC3554605

[17] The Plant List. Version 1.1. [Internet]. 2013. Accessed 2015 Jul 2. Available: http://www.theplantlist.org/.

[18] Index Fungorum. [Internet]. 2015. Accessed 2015 Jul 2. Available: http://www.indexfungorum.org/.

[19] World Spider Catalog. Version 16.5. [Internet]. Natural History Museum Bern. 2015. Accessed 2015 Jul 2. Available: http://wsc.nmbe.ch.

[20] Lepage D. Avibase. [Internet]. 2015. Accessed 2015 Jul 2. Available: http://avibase.bsc-eoc.org/avibase.jsp?lang=EN.

[21] Lepage D, Vaidya G, Guralnick RP. Avibase–a database system for managing and organizing taxonomic concepts. ZooKeys 2014; 420:117–135. 10.3897/zookeys.420.7089 25061375 PMC4109484

[22] Froese R, Pauly D. Editors. FishBase. [Internet]. 2015. Accessed 2015 Jul 2. Available: www.fishbase.org.

[23] Eschmeyer WN (ed). Catalog of Fishes: genera, species, referenced. [Internet]. 2015. Accessed 2015 Jul 2. Available: http://researcharchive.calacademy.org/research/ichthyology/catalog/fishcatmain.asp.

[24] Costello MJ, Bouchet P, Boxshall G, Fauchald K, Gordon D, Hoeksema BW, et al Global Coordination and Standardisation in Marine Biodiversity through the World Register of Marine Species (WoRMS) and Related Databases. PLoS ONE 2013; 8(1): e51629 10.1371/journal.pone.0051629 23505408 PMC3541386

[25] WoRMS Editorial Board. World Register of Marine Species. [Internet]. 2015. Accessed 2015 Jul 2. Available: http://www.marinespecies.org.

[26] Uetz P, Hošek J (eds.). The Reptile Database. [Internet]. 2015. Accessed 2015 Jul 2. Available: http://www.reptile-database.org.

[27] AmphibiaWeb: Information on amphibian biology and conservation. [Internet]. 2015. Berkeley, California: AmphibiaWeb. Accessed 2015 Jul 2. Available: http://amphibiaweb.org/.

[28] Wilson DE, Reeder DM. Mammal Species of the World—A Taxonomic and Geographic Reference (Third ed.). Baltimore, Maryland: Johns Hopkins University Press/Bucknell University 2005 pp. 2,142. ISBN 978-0-8018-8221-0.

[29] Wilson DE, Reeder DM, John Hopkins University Press. Mammal Species of the World, 3rd edition. Press. 2015. [Internet]. Accessed 2015 Jul 2. Available: http://vertebrates.si.edu/msw/mswcfapp/msw/index.cfm.

[30] Integrated Taxonomic Information System (ITIS). [Internet]. 2015. Accessed 2015 Jul 2. Available: http://www.itis.gov.

[31] Roskov Y, Abucay L, Orrell T, Nicolson D, Kunze T, Flann C, et al., eds. Species 2000 & ITIS Catalogue of Life, 30th July 2015. [Internet]. Species 2000: Naturalis, Leiden, the Netherlands. 2015. ISSN 2405-8858. Accessed 2015 Jul 2. Available: www.catalogueoflife.org/col.

[32] Global Biodiversity Information Facility (GBIF) Secretariat. GBIF Backbone Taxonomy. [Internet]. 2013. Accessed 2015 Jul 2. Available: http://www.gbif.org/dataset/d7dddbf4-2cf0-4f39-9b2a-bb099caae36c.

[33] Vanden Berghe E, Coro G, Bailly N, Fiorellato F, Aldemita C, Ellenbroek A, et al Retrieving taxa names from large biodiversity data collections using a flexible matching workflow. Ecol Inform 2015; 28: 29–41.

[34] Gaiji S, Chavan V, Ariño AH, Otegui J, Hobern D, Sood R, et al Content Assessment of the Primary Biodiversity Data Published through GBIF Network: status, challenges and potentials. Biodiversity Informatics 2013; 8: 94–172.

[35] Peng T, Li L, Kennedy J. A comparison of techniques for name matching. GSTF Journal on Computing (JoC) 2012; 2 (1):55–61.

[36] Constable H, Guralnick R, Wieczorek J, Spencer CL, Peterson AT. VertNet: A new model for biodiversity data sharing. PLoS Biol 2010; 8:1–4. 10.1371/journal.pbio.1000309 PMC2821892 20169109

[37] VertNet. [Internet]. 2015. Accessed 2015 Jul. Available: http://vertnet.org.

[38] Robertson T, Döring M, Guralnick R, Bloom D, Wieczorek J, Braak K, et al The GBIF Integrated Publishing Toolkit: Facilitating the Efficient Publishing of Biodiversity Data on the Internet. PLoS ONE 2014; 9(8): e102623 10.1371/journal.pone.0102623 25099149 PMC4123864

[39] Chapman AD. Principles and methods of data cleaning–primary species and species-occurrence data, version 1.0. Report for the Global Biodiversity Information Facility, Copenhagen; 2005.

[40] Damerau F. A technique for computer detection and correction of spelling errors. Commun. ACM 1964; 7:171–176.

[41] International Commission on Zoological Nomenclature. International Code of Zoological Nomenclature. 4th Ed. International Trust for Zoological Nomenclature, London; 1999. Accessed 2015 Jul 2. Available: http://www.nhm.ac.uk/hosted-sites/iczn/code/.

[42] Kluyver TA, Osborne CP. Taxonome: a software package for linking biological species data. Ecol Evol 2013; 3(5): 1262–1265. 10.1002/ece3.529 23762512 PMC3678480

[43] Schuh RT. The Linnean System and its 250-year persistence. Bot Rev 2003; 69(1):59–78.

[44] Berendsohn WG. The Concept of "Potential Taxa" in Databases. Taxon 1995; 44(2): 207–212.

[45] Gill FB. Species taxonomy of birds: Which null hypothesis?. The Auk 2014; 131(2):150–161.

[46] VertNet IPT. [Internet]. 2015. Accessed 2015 Apr. Available: http://ipt.vertnet.org:8080/ipt/.

[47] Wieczorek J. VertNet Darwin Core Data Migrator Toolkit. GitHub repository. [Internet]. 2015a. Available: https://github.com/vertnet/toolkit.

[48] Wieczorek J. VertNet Darwin Core Vocabularies. GitHub repository. [Internet]. 2015b. Available: https://github.com/tucotuco/DwCVocabs.

[49] R Core Team. R: A language and environment for statistical computing R Foundation for Statistical Computing, Vienna, Austria 2015 ISBN 3-900051-07-0. Available: http://www.R-project.org/.

[50] Burnham KP, Anderson DR. Model selection and multimodel inference: a practical information-theoretic approach. New York: Springer-Verlag; 2002. 488p.

[51] Bartoń K. MuMIn: Multi-Model Inference Package. 2015. Available: https://cran.r-project.org/web/packages/MuMIn/index.html.

[52] Beaujean AA. R Package for Baylor University Educational Psychology Quantitative Courses. 2015. Available: https://cran.r-project.org/web/packages/BaylorEdPsych/index.html.

[53] Fox J. Effect Displays in R for Generalised Linear Models. J Stat Softw 2003; 8(15): 1–27. Package Available: https://cran.r-project.org/web/packages/effects/index.html.

[54] Alroy J. How many named species are valid? Proc. Natl. Acad. Sci. U. S. A. 2002; 99:3706–3711. 11891342 10.1073/pnas.062691099 PMC122588

[55] Padial JM, De la Riva I. Taxonomic inflation and the stability of species lists: the perils of ostrich’s behavior. Syst Biol 2006; 55(5):859–867. 17060206 10.1080/1063515060081588

[56] Costello MJ, Wieczorek J. Best practice for biodiversity data management and publication. Biol Cons 2013; 173 10.1016/j.biocon.2013.10.018

[57] Rees T. Taxamatch, an Algorithm for Near (‘Fuzzy’) Matching of Scientific Names in Taxonomic Databases. PLoS ONE 2014; 9(9): e107510 10.1371/journal.pone.0107510 25247892 PMC4172526

[58] Duarte M, Guerrero PC, Carvallo G, and Bustamante RO. Conservation network design for endemic cacti under taxonomic uncertainty. Biological Conservation 2014; 176:236–242. Available: 10.1016/j.biocon.2014.05.028.

