# Supplementary figures and images for: A Standardized Reference Data Set for Vertebrate Taxon Name Resolution
Source: PLoS One. 2016 Jan 13;11(1):e0146894. doi: 10.1371/journal.pone.0146894 (PMC4711887; doi:10.1371/journal.pone.0146894)

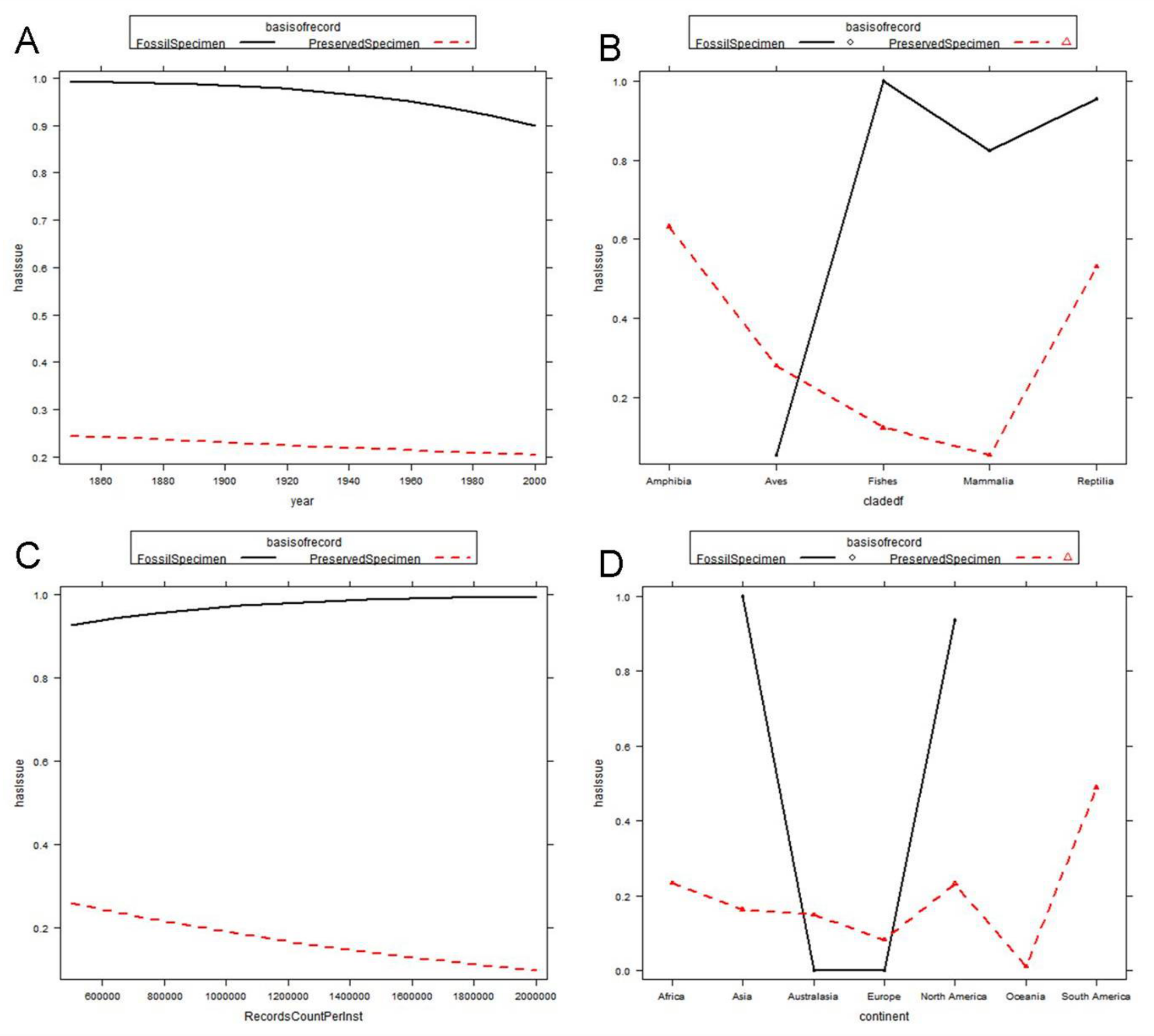

Supplement: S1 Fig — Effect of the interaction of the variables basisOfRecord, Geographic region (shown as “continent”), Clade (shown as “cladedf”), Volume of records shared by Institution (shown as “RecordsCountPerInst”) and year on the probability of occurrence of at least one of the following issues: Synonymy, Misspelling, Conceptual error, Format Error. Effects calculated through logit GLM, with binomial response using the R package “effects”. A: basisOfRecord:year; B: basisOfRecord:Clade; C: basisOfRecord:Volume of records shared; D: basisOfRecord:Geographic region. (TIF) [file pone.0146894.s001.tif]

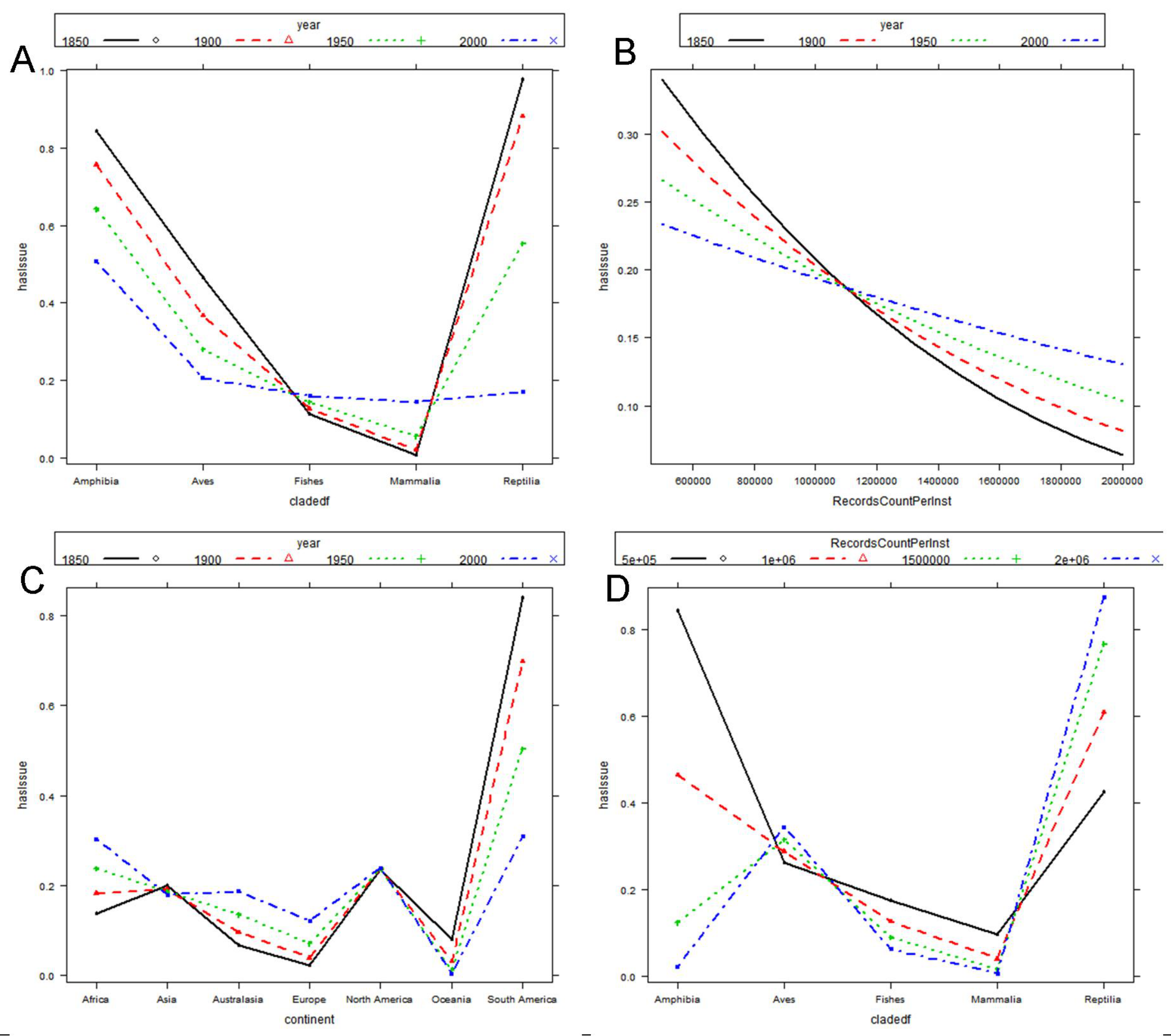

Supplement: S2 Fig — Effect of the interaction of the variables basisOfRecord, Geographic region (shown as “continent”), Clade (shown as “cladedf”), Volume of records shared by Institution (shown as “RecordsCountPerInst”) and year on the probability of occurrence of at least one of the following issues: Synonymy, Misspelling, Conceptual error, Format Error. Effects calculated through logit GLM, with binomial response using the R package “effects”. A: Year:Clade; B: Year:Volume of records shared; C: Year:Geographic region; D: Clade:Volume of records shared. (TIF) [file pone.0146894.s002.tif]

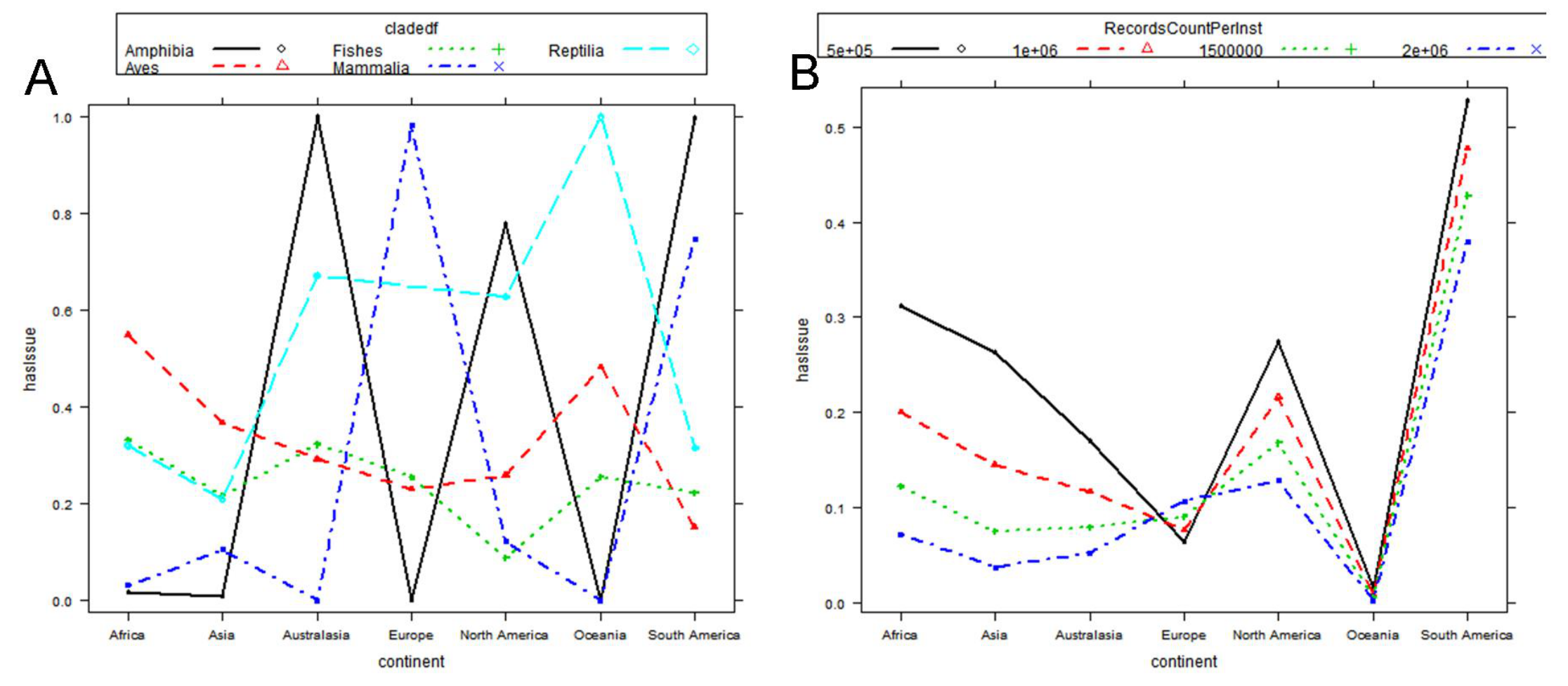

Supplement: S3 Fig — Effect of the interaction of the variables basisOfRecord, Geographic region (shown as “continent”), Clade (shown as “cladedf”), Volume of records shared by Institution (shown as “RecordsCountPerInst”) and year on the probability of occurrence of at least one of the following issues: Synonymy, Misspelling, Conceptual error, Format Error. Effects calculated through logit GLM, with binomial response using the R package “effects”. A: Clade:Geographic region; B: Volume of records shared:Geographic region. (TIF) [file pone.0146894.s003.tif]
