# Supplementary material for: A Standardized Reference Data Set for Vertebrate Taxon Name Resolution
Source: PLoS One. 2016 Jan 13;11(1):e0146894. doi: 10.1371/journal.pone.0146894 (PMC4711887; doi:10.1371/journal.pone.0146894)
Supplement: S1 File — (DOC) [file pone.0146894.s004.doc]

**S1 File. Detailed VertNet Names Data Acquisition**

1. AGGREGATION OF NON-MIGRATED NAMES

The 18 April 2015 snapshot of 17,412,547 VertNet records was uploaded into a Google BigQuery table (vertnet_latest) for easy filtering and extraction of subsets. Then a summary table of taxa by data set was extracted from BigQuery using the following query:

SELECT icode, collectioncode, gbifdatasetid, scientificname, genus, subgenus, specificepithet, infraspecificepithet, scientificnameauthorship, migrator, count(*) as reps FROM [dumps.vertnet_latest] group by icode, collectioncode, gbifdatasetid, scientificname, genus, subgenus, specificepithet, infraspecificepithet, scientificnameauthorship, migrator

This summary was saved in a new table called vn_scinames_by_dataset and contained 892128 distinct name combinations. This new table was used to extract a subset of non-migrated taxon combinations using the following query:

SELECT scientificname, genus, subgenus, specificepithet, infraspecificepithet, scientificnameauthorship, sum(reps) as totaloccurrences FROM [dumps.vn_scinames_by_dataset] where migrator is null or migrator='no migrator' group by scientificname, genus, subgenus, specificepithet, infraspecificepithet, scientificnameauthorship

This summary was saved in a new table called vn_scinames_from_selfhosted and contained 304127 distinct combinations. The following query was run to extract complementary data for migrated data sets (for reference only - these data were not used to construct this test data set):

SELECT scientificname, genus, subgenus, specificepithet, infraspecificepithet, scientificnameauthorship, sum(reps) as totaloccurrences FROM [dumps.vn_scinames_by_dataset] where migrator like '%-%' group by scientificname, genus, subgenus, specificepithet, infraspecificepithet, scientificnameauthorship

This summary was saved in a new table called vn_scinames_from_migrators and contained 158250 distinct combinations after migration (compared to 399173 combinations before migration, determined separately).

The vn_scinames_from_selfhosted table was saved to Google Cloud storage as a gzipped CSV file vn_scinames_from_selfhosted.csv, downloaded, and unzipped. The resulting CSV file was loaded into a table vn_names_from_selfhosted in a Microsoft Access database using the same database schema as in BigQuery.

The table vn_names_from_selfhosted was duplicated with the same data to a new table vn_names_all. The table vn_names_from_migrators was created without data using the same structure as vn_names_from_selfhosted.

1. AGGREGATION OF MIGRATED NAMES

Each customized VertNet migrator contains a table SimpleDwC-verbatim into which the verbatim source data are loaded before further processing. For each migrated data set, the distinct combinations of institutionCode, scientificName, genus, subgenus, specificEpithet, infraspecificEpithet, scientificNameAuthorship, count(*) as reps were extracted from the migrator's table SimpleDwC-verbatim and appended to the table vn_names_from_migrators.

When all migrators had been processed and the data added to the vn_names_from_migrators table, the distinct combinations of scientificName, genus, subgenus, specificEpithet, infraspecificEpithet, scientificNameAuthorship, and reps were extracted and appended to table vn_names_all.

1. SAMPLING OF 1000 RANDOM NAME RECORDS

At this point the table vn_names_all contained the full set of verbatim name combinations of those fields across all of published VertNet as of 18 April 2014. The table vn_names_distinct was created empty with the same structure as table vn_names_all. The field 'id' was added to table vn_names_distinct of type Autonumber with random new long integer values. This enabled each new record added to the table to receive a distinct random id.

The table vn_names_distinct was filled by appending from a view defined as

SELECT scientificName, genus, subgenus, specificEpithet, infraspecificEpithet, scientificNameAuthorship, sum(totaloccurrences) as totaloccurrences FROM vn_names_all group by scientificName, genus, subgenus, specificEpithet, infraspecificEpithet, scientificNameAuthorship

This resulted in a completed table vn_names_distinct with 522163 distinct combinations of taxon records from across all of VertNet as of 18 April 2015. This table constitutes the raw material from which to draw the random subset of 1000 records for this test data set.

The table 1000VNNames was created with the fields described in Table 1S, along with various auxiliary fields to help with the management of processing subsets of the test data. To populate the table 1000VNNames with 1000 names, the id of the 1000th record when sorted in ascending order was found and used as a filter to select the first 1000 id-sorted names where the ids were generated randomly as described above. The table 1000VNNames was then populated by appending records from the following query:

SELECT id, scientificname, genus, subgenus, specificepithet, infraspecificepithet, scientificnameauthorship from TABLE vn_names_distinct INTO TABLE 1000VNNames where id<= {value of 1000th sorted id}

During the course of name resolution and vetting, 34 records from among the original 1000 were found to be for non-vertebrates (VertNet does indeed have some non-vertebrate records from some sources for various reasons). These were replaced using the same method to bring the final count of the test data set to 1000 records from vertebrates.

A field called constructedscientificname, to facilitate valid name resolution, was populated with the space-separated concatenation of genus, subgenus, specificEpithet, infraspecificEpithet, and scientificNameAuthorship. In the cases where only the infraspecificEpithet field in the record was populated with data, the _constructedscientificname was left blank.

A field called scientificnameplus was created to provide a corollary to the constructedscientificname based on the scientificName field. Inspection of the data set revealed that some verbatim records relied on a combination of scientificName and infraspecificEpithet to produce the full scientific name, apparently taking the scientificName field to be populated with nothing more specific than a binomial. Thus, for records where scientificName and infraspecificEpithet were both populated and the scientificName did not contain an infraspecificEpithet, the field scientificnameplus was constructed from the space-separated concatenation of the two, otherwise it was populated by only the value in the scientificName field.

A field called who was created to track the researcher to whom primary responsibility for vetting was assigned. The first 500 of the 1000 name combinations were assigned to one vetter and the remaining 500 were assigned to the other vetter. Subsets of the table 1000VNNames were extracted into template Microsoft Excel spreadsheets in which the vetters conducted their research independently.
