# Supplementary material for: A Standardized Reference Data Set for Vertebrate Taxon Name Resolution
Source: PLoS One. 2016 Jan 13;11(1):e0146894. doi: 10.1371/journal.pone.0146894 (PMC4711887; doi:10.1371/journal.pone.0146894)
Supplement: S2 File — (DOC) [file pone.0146894.s005.doc]

**S2 File. Supplementary References**

AMNH Bird Collection. American Museum of Natural History. [Internet]. 2015. [Accessed 2015 Apr 18]. Available from: http://ipt.vertnet.org:8080/ipt/resource.do?r=amnh_birds

AMNH Herpetology Collection. American Museum of Natural History. [Internet]. 2015. [Accessed 2015 Apr 18]. Available from: http://ipt.vertnet.org:8080/ipt/resource.do?r=amnh_herps

AMNH Mammal Collection. American Museum of Natural History. [Internet]. 2015. [Accessed 2015 Apr 18]. Available from: http://ipt.vertnet.org:8080/ipt/resource.do?r=amnh_mammals

ANSP Herpetology Collection. Academy of Natural Sciences of Philadelphia. [Internet]. 2015. [Accessed 2015 Apr 18]. Available from: http://phylo.ansp.org:8080/ipt/resource.do?r=ansp_hrp

ANSP Ichthyology Collection. Academy of Natural Sciences of Philadelphia. [Internet]. 2015. [Accessed 2015 Apr 18]. Available from: http://phylo.ansp.org:8080/ipt/resource.do?r=ansp_ich

ANSP Mammalogy Collection. Academy of Natural Sciences of Philadelphia. [Internet]. 2015. [Accessed 2015 Apr 18]. Available from: http://phylo.ansp.org:8080/ipt/resource.do?r=ansp_mam

ANSP Ornithology Collection. Academy of Natural Sciences of Philadelphia. [Internet]. 2015. [Accessed 2015 Apr 18]. Available from: http://phylo.ansp.org:8080/ipt/resource.do?r=ansp_orn

ASNHC Mammalogy Collection. Angelo State Natural History Collections, Angelo State University. [Internet]. 2015. [Accessed 2015 Apr 18]. Available from: http://ipt.vertnet.org:8080/iptstrays/resource.do?r=angelo_mammals

ASNHC Ornithology Collection. Angelo State Natural History Collections, Angelo State University. [Internet]. 2015. [Accessed 2015 Apr 18]. Available from: http://ipt.vertnet.org:8080/iptstrays/resource.do?r=angelo_birds

AUM Fish Collection. Auburn University Museum of Natural History. [Internet]. 2015. [Accessed 2015 Apr 18]. Available from: http://ipt.vertnet.org:8080/ipt/resource.do?r=aum_fish

Australian Museum provider for OZCAM. Australian Museum. [Internet]. 2015. [Accessed 2015 Apr 18]. Available from: http://collections.ala.org.au/public/show/dr340

Australian National Wildlife Collection provider for OZCAM. Australian National Wildlife Collection. [Internet]. 2015. [Accessed 2015 Apr 18]. Available from: http://collections.ala.org.au/public/show/dr341

Aves Tanzanian collection at the Natural History Museum of Denmark (SNM). Zoological Museum, Natural History Museum of Denmark. [Internet]. 2015. [Accessed 2015 Apr 18]. Available from: http://danbif.au.dk/ipt/resource.do?r=aves_tanza

Borror Lab of Bioacoustics (BLB). Ohio State University. [Internet]. 2015. [Accessed 2015 Apr 18]. Available from: http://hymfiles.biosci.ohio-state.edu:8080/ipt/resource.do?r=blb

BPBM collections. Bernice P. Bishop Museum. [Internet]. 2015. [Accessed 2015 Apr 18]. Available from: http://ipt.vertnet.org:8080/ipt/resource.do?r=bpbm

BYU Herpetology Collection. Monte L. Bean Museum, Brigham Young University. [Internet]. 2015. [Accessed 2015 Apr 18]. Available from: http://www.gbif.org/dataset/847aa410-f762-11e1-a439-00145eb45e9a

CAS Herpetology (HERP). California Academy of Sciences. [Internet]. 2015. [Accessed 2015 Apr 18]. Available from: http://ipt.calacademy.org:8080/ipt/resource.do?r=herp

CAS Ichthyology (ICH). California Academy of Sciences. [Internet]. 2015. [Accessed 2015 Apr 18]. Available from: http://ipt.calacademy.org:8080/ipt/resource.do?r=ich

CAS Mammalogy (MAM). California Academy of Sciences. [Internet]. 2015. [Accessed 2015 Apr 18]. Available from: http://ipt.calacademy.org:8080/ipt/resource.do?r=mam

CAS Ornithology (ORN). California Academy of Sciences. [Internet]. 2015. [Accessed 2015 Apr 18]. Available from: http://ipt.calacademy.org:8080/ipt/resource.do?r=orn

CCBER Mammalogy Collection. Cheadle Center for Biodiversity and Ecological Restoration. [Internet]. 2015. [Accessed 2015 Apr 18]. Available from: http://ipt.vertnet.org:8080/ipt/resource.do?r=ccber_mammals

CHAS Vertebrate Collections. Chicago Academy of Sciences. [Internet]. 2015. [Accessed 2015 Apr 18]. Available from: http://ipt.vertnet.org:8080/ipt/resource.do?r=chas_verts

CM Birds Collection. Carnegie Museum of Natural History. [Internet]. 2015. [Accessed 2015 Apr 18]. Available from: http://ipt.vertnet.org:8080/ipt/resource.do?r=cm_birds

CM Herps Collection. Carnegie Museum of Natural History. [Internet]. 2015. [Accessed 2015 Apr 18]. Available from: http://ipt.vertnet.org:8080/ipt/resource.do?r=cm_herps

Cowan Tetrapod Collection - Birds (UBCBBM). University of British Columbia Beaty Biodiversity Museum. [Internet]. 2015. [Accessed 2015 Apr 18]. Available from: http://ipt.vertnet.org:8080/ipt/resource.do?r=ubc_bbm_ctc_birds

Cowan Tetrapod Collection - Herpetology (UBCBBM). University of British Columbia Beaty Biodiversity Museum. [Internet]. 2015. [Accessed 2015 Apr 18]. Available from: http://ipt.vertnet.org:8080/ipt/resource.do?r=ubc_bbm_ctc_herps

CRCM Vertebrate Collection. Charles R. Conner Museum. [Internet]. 2015. [Accessed 2015 Apr 18]. Available from: http://ipt.vertnet.org:8080/ipt/resource.do?r=crcm_verts

CSIRO Ichthyology provider for OZCAM. Commonwealth Scientific and Industrial Research Organization. [Internet]. 2015. [Accessed 2015 Apr 18]. Available from: http://collections.ala.org.au/public/show/dr349

CSUC Mammalogy Collection. California State University, Chico Vertebrate Museum. [Internet]. 2015. [Accessed 2015 Apr 18]. Available from: http://ipt.vertnet.org:8080/ipt/resource.do?r=csuc_verts

CUMV Bird Collection (Arctos). Cornell University Museum of Vertebrates. [Internet]. 2015. [Accessed 2015 Apr 18]. Available from: http://ipt.vertnet.org:8080/ipt/resource.do?r=cumv_bird

CUMV Fish Collection (Arctos). Cornell University Museum of Vertebrates. [Internet]. 2015. [Accessed 2015 Apr 18]. Available from: http://ipt.vertnet.org:8080/ipt/resource.do?r=cumv_fish

CUMV Mammal Collection (Arctos). Cornell University Museum of Vertebrates. [Internet]. 2015. [Accessed 2015 Apr 18]. Available from: http://ipt.vertnet.org:8080/ipt/resource.do?r=cumv_mamm

CUMV Reptile Collection (Arctos). Cornell University Museum of Vertebrates. [Internet]. 2015. [Accessed 2015 Apr 18]. Available from: http://ipt.vertnet.org:8080/ipt/resource.do?r=cumv_rept

DMNH Bird Collection. Delaware Museum of Natural History. [Internet]. 2015. [Accessed 2015 Apr 18]. Available from: http://ipt.vertnet.org:8080/ipt/resource.do?r=dmnh_birds

DMNS Bird Collection (Arctos). Denver Museum of Nature & Science. [Internet]. 2015. [Accessed 2015 Apr 18]. Available from: http://ipt.vertnet.org:8080/ipt/resource.do?r=dmns_bird

DMNS Mammal Collection (Arctos). Denver Museum of Nature & Science. [Internet]. 2015. [Accessed 2015 Apr 18]. Available from: http://ipt.vertnet.org:8080/ipt/resource.do?r=dmns_mamm

FLMNH Herpetology. Florida Museum of Natural History, University of Florida. [Internet]. 2015. [Accessed 2015 Apr 18]. Available from: http://ipt.flmnh.ufl.edu:8080/ipt/resource.do?r=herpetology

FLMNH Ichthyology. Florida Museum of Natural History, University of Florida. [Internet]. 2015. [Accessed 2015 Apr 18]. Available from: http://ipt.flmnh.ufl.edu:8080/ipt/resource.do?r=ichthyology

FLMNH Mammals. Florida Museum of Natural History, University of Florida. [Internet]. 2015. [Accessed 2015 Apr 18]. Available from: http://ipt.flmnh.ufl.edu:8080/ipt/resource.do?r=mammals

FLMNH Ornithology. Florida Museum of Natural History, University of Florida. [Internet]. 2015. [Accessed 2015 Apr 18]. Available from: http://ipt.vertnet.org:8080/iptstrays/resource.do?r=flmnh_birds

FMNH (Zoology) Amphibian and Reptile Collection. Field Museum of Natural History. [Internet]. 2015. [Accessed 2015 Apr 18]. Available from: http://fmipt.fieldmuseum.org:8080/ipt/resource.do?r=fmnh_herps

FMNH (Zoology) Bird Collection. Field Museum of Natural History. [Internet]. 2015. [Accessed 2015 Apr 18]. Available from: http://fmipt.fieldmuseum.org:8080/ipt/resource.do?r=fm_birds

FMNH (Zoology) Bird Egg Collection. Field Museum of Natural History. [Internet]. 2015. [Accessed 2015 Apr 18]. Available from: http://fmipt.fieldmuseum.org:8080/ipt/resource.do?r=fm_birds_eggs

FMNH (Zoology) Fish Collection. Field Museum of Natural History. [Internet]. 2015. [Accessed 2015 Apr 18]. Available from: http://fmipt.fieldmuseum.org:8080/ipt/resource.do?r=fmnh_fishes

FMNH (Zoology) Mammal Collection. Field Museum of Natural History. [Internet]. 2015. [Accessed 2015 Apr 18]. Available from: http://fmipt.fieldmuseum.org:8080/ipt/resource.do?r=fmnh_mammals

KSTC Mammals. Schmidt Museum of Natural History, Emporia State University. [Internet]. 2015. [Accessed 2015 Apr 18]. Available from: http://ipt.vertnet.org:8080/ipt/resource.do?r=kstc_schmidt_mammals

KUBI Herpetology Collection. KU Biodiversity Institute, University of Kansas. [Internet]. 2015. [Accessed 2015 Apr 18]. Available from: http://ipt.nhm.ku.edu/ipt/resource.do?r=kubi_herps

KUBI Ichthyology Collection. KU Biodiversity Institute, University of Kansas. [Internet]. 2015. [Accessed 2015 Apr 18]. Available from: http://ipt.nhm.ku.edu/ipt/resource.do?r=kubi_ichthyology

KUBI Ichthyology Collection. KU Biodiversity Institute, University of Kansas. [Internet]. 2015. [Accessed 2015 Apr 18]. Available from: http://ipt.nhm.ku.edu/ipt/resource.do?r=kubi_ichthyology_tissue

KUBI Mammalogy Collection. KU Biodiversity Institute, University of Kansas. [Internet]. 2015. [Accessed 2015 Apr 18]. Available from: http://ipt.nhm.ku.edu/ipt/resource.do?r=kubi_mammals

KUBI Vertebrate Paleontology Collection. KU Biodiversity Institute, University of Kansas. [Internet]. 2015. [Accessed 2015 Apr 18]. Available from: http://ipt.nhm.ku.edu/ipt/resource.do?r=kubi_vertpaleo

LACM Vertebrate Collection. Natural History Museum of Los Angeles County. [Internet]. 2015. [Accessed 2015 Apr 18]. Available from: http://ipt.vertnet.org:8080/ipt/resource.do?r=lacm_verts

MMNH Bird Collection. Bell Museum of Natural History. [Internet]. 2015. [Accessed 2015 Apr 18]. Available from: http://ipt.vertnet.org:8080/ipt/resource.do?r=mmnh_birds

MNHN Birds collection (ZO). Museum national d'Histoire naturelle, Paris. [Internet]. 2015. [Accessed 2015 Apr 18]. Available from: http://collections.mnhn.fr/ipt/resource.do?r=mnhn-zo

MNHN Fishes collection (IC). Museum national d'Histoire naturelle, Paris. [Internet]. 2015. [Accessed 2015 Apr 18]. Available from: http://collections.mnhn.fr/ipt/resource.do?r=mnhn-ic

MNHN Mammals collection (ZM). Museum national d'Histoire naturelle, Paris. [Internet]. 2015. [Accessed 2015 Apr 18]. Available from: http://collections.mnhn.fr/ipt/resource.do?r=mnhn-zm

MNHN Reptiles and Amphibians collection (RA). Museum national d'Histoire naturelle, Paris. [Internet]. 2015. [Accessed 2015 Apr 18]. Available from: http://collections.mnhn.fr/ipt/resource.do?r=mnhn-ra

MSB Bird Collection (Arctos). Museum of Southwestern Biology, University of New Mexico, Albuquerque. [Internet]. 2015. [Accessed 2015 Apr 18]. Available from: http://ipt.vertnet.org:8080/ipt/resource.do?r=msb_bird

MSB Mammal Collection (Arctos). Museum of Southwestern Biology, University of New Mexico, Albuquerque. [Internet]. 2015. [Accessed 2015 Apr 18]. Available from: http://ipt.vertnet.org:8080/ipt/resource.do?r=msb_mamm

MSU Mammalogy, Ornithology and Vertebrate Paleontology Collections. Michigan State University. [Internet]. 2015. [Accessed 2015 Apr 18]. Available from: http://ipt.vertnet.org:8080/ipt/resource.do?r=msu_mammals_birds_vertpaleo

MSUM Ichthyology and Herpetology Collections. Michigan State University Museum. [Internet]. 2015. [Accessed 2015 Apr 18]. Available from: http://ipt.vertnet.org:8080/ipt/resource.do?r=msum_fish_herps

Museum of Comparative Zoology (MCZ). Harvard University. [Internet]. 2015. [Accessed 2015 Apr 18]. Available from: http://digir.mcz.harvard.edu/ipt/resource.do?r=mcz_subset_for_vertnet

MVZ Bird Collection (Arctos). Museum of Vertebrate Zoology, University of California, Berkeley. [Internet]. 2015. [Accessed 2015 Apr 18]. Available from: http://ipt.vertnet.org:8080/ipt/resource.do?r=mvz_bird

MVZ Bird Observations (Arctos). Museum of Vertebrate Zoology, University of California, Berkeley. [Internet]. 2015. [Accessed 2015 Apr 18]. Available from: http://ipt.vertnet.org:8080/ipt/resource.do?r=mvzobs_bird

MVZ Egg and Nest Collection (Arctos). Museum of Vertebrate Zoology, University of California, Berkeley. [Internet]. 2015. [Accessed 2015 Apr 18]. Available from: http://ipt.vertnet.org:8080/ipt/resource.do?r=mvz_egg

MVZ Herp Collection (Arctos). Museum of Vertebrate Zoology, University of California, Berkeley. [Internet]. 2015. [Accessed 2015 Apr 18]. Available from: http://ipt.vertnet.org:8080/ipt/resource.do?r=mvz_herp

MVZ Mammal Collection (Arctos). Museum of Vertebrate Zoology, University of California, Berkeley. [Internet]. 2015. [Accessed 2015 Apr 18]. Available from: http://ipt.vertnet.org:8080/ipt/resource.do?r=mvz_mammal

NCSM Birds Collection. North Carolina Museum of Natural Sciences. [Internet]. 2015. [Accessed 2015 Apr 18]. Available from: http://ipt.vertnet.org:8080/ipt/resource.do?r=ncsm_birds

NCSM Fishes Collection. North Carolina Museum of Natural Sciences. [Internet]. 2015. [Accessed 2015 Apr 18]. Available from: http://ipt.vertnet.org:8080/ipt/resource.do?r=ncsm_fishes

NCSM Herpetology Collection. North Carolina Museum of Natural Sciences. [Internet]. 2015. [Accessed 2015 Apr 18]. Available from: http://ipt.vertnet.org:8080/ipt/resource.do?r=ncsm_herps

NCSM Vertebrate Paleontology Collection. North Carolina Museum of Natural Sciences. [Internet]. 2015. [Accessed 2015 Apr 18]. Available from: http://ipt.vertnet.org:8080/ipt/resource.do?r=ncsm_vertpaleo

NMMNH Mammal. New Mexico Museum of Natural History and Science. [Internet]. 2015. [Accessed 2015 Apr 18]. Available from: http://ipt.vertnet.org:8080/ipt/resource.do?r=nmmnh_mammals

NMNH Amphibians & Reptiles. National Museum of Natural History, Smithsonian Institution. [Internet]. 2015. [Accessed 2015 Apr 18]. Available from: http://collections.mnh.si.edu/ipt/resource.do?r=nmnhdwca

NMNH Birds. National Museum of Natural History, Smithsonian Institution. [Internet]. 2015. [Accessed 2015 Apr 18]. Available from: http://collections.mnh.si.edu/ipt/resource.do?r=nmnhdwca

NMNH Fishes. National Museum of Natural History, Smithsonian Institution. [Internet]. 2015. [Accessed 2015 Apr 18]. Available from: http://collections.mnh.si.edu/ipt/resource.do?r=nmnhdwca

NMNH Mammals. National Museum of Natural History, Smithsonian Institution. [Internet]. 2015. [Accessed 2015 Apr 18]. Available from: http://collections.mnh.si.edu/ipt/resource.do?r=nmnhdwca

NMNH Paleobiology (vertebrates). National Museum of Natural History, Smithsonian Institution. [Internet]. 2015. [Accessed 2015 Apr 18]. Available from: http://collections.mnh.si.edu/ipt/resource.do?r=nmnhdwca

Northern Territory Museum and Art Gallery provider for OZCAM. Northern Territory and Art Museum. [Internet]. 2015. [Accessed 2015 Apr 18]. Available from: http://collections.ala.org.au/public/show/dr343

NPL collections. The Non-vertebrate Paleontology Laboratory at The University of Texas at Austin. [Internet]. 2015. [Accessed 2015 Apr 18]. Available from: http://ipt.vertnet.org:8080/ipt/resource.do?r=npl_nonvertpaleo

NYSM Mammals. New York State Museum. [Internet]. 2015. [Accessed 2015 Apr 18]. Available from: http://ipt.vertnet.org:8080/ipt/resource.do?r=nysm_mammals

OMNH Amphibian Specimens. Sam Noble Oklahoma Museum of Natural History. [Internet]. 2015. [Accessed 2015 Apr 18]. Available from: http://iptsnomnh.cloudapp.net:8080/ipt/resource.do?r=amphibian

OMNH Eggs Specimens. Sam Noble Oklahoma Museum of Natural History. [Internet]. 2015. [Accessed 2015 Apr 18]. Available from: http://iptsnomnh.cloudapp.net:8080/ipt/resource.do?r=eggs

OMNH Tissues Specimens. Sam Noble Oklahoma Museum of Natural History. [Internet]. 2015. [Accessed 2015 Apr 18]. Available from: http://iptsnomnh.cloudapp.net:8080/ipt/resource.do?r=tissue

OSU Ichthyology Collection. Oregon State University. [Internet]. 2015. [Accessed 2015 Apr 18]. Available from: http://pontos.fwl.oregonstate.edu:8080/ipt/resource.do?r=oregonstate_fish

OSUM Fish Division. Ohio State University. [Internet]. 2015. [Accessed 2015 Apr 18]. Available from: http://hymfiles.biosci.ohio-state.edu:8080/ipt/resource.do?r=osum-fish

OSUM Tetrapod Division - Bird Collection. Ohio State University. [Internet]. 2015. [Accessed 2015 Apr 18]. Available from: http://hymfiles.biosci.ohio-state.edu:8080/ipt/resource.do?r=osum-birds

OSUM Tetrapod Division - Reptile Collection. Ohio State University. [Internet]. 2015. [Accessed 2015 Apr 18]. Available from: http://hymfiles.biosci.ohio-state.edu:8080/ipt/resource.do?r=osum-reptiles

PMNS Natural History Collections. Perot Museum of Nature and Science. [Internet]. 2015. [Accessed 2015 Apr 18]. Available from: http://ipt.vertnet.org:8080/ipt/resource.do?r=perot_verts

PSM Vertebrates Collection. James R. Slater Museum of Natural History. [Internet]. 2015. [Accessed 2015 Apr 18]. Available from: http://ipt.vertnet.org:8080/ipt/resource.do?r=psm_verts

Queen Victoria Museum Art Gallery provider for OZCAM. Queen Victoria Museum and Art Gallery. [Internet]. 2015. [Accessed 2015 Apr 18]. Available from: http://collections.ala.org.au/public/show/dr345

Queensland Museum provider for OZCAM. Queensland Museum. [Internet]. 2015. [Accessed 2015 Apr 18]. Available from: http://collections.ala.org.au/public/show/dr344

ROM Herpetology Collection. Royal Ontario Museum. [Internet]. 2015. [Accessed 2015 Apr 18]. Available from: http://gbif.rom.on.ca:8180/ipt/resource.do?r=herps

ROM Ichthyology Collection. Royal Ontario Museum. [Internet]. 2015. [Accessed 2015 Apr 18]. Available from: http://gbif.rom.on.ca:8180/ipt/resource.do?r=fishes

ROM Mammalogy Collection. Royal Ontario Museum. [Internet]. 2015. [Accessed 2015 Apr 18]. Available from: http://gbif.rom.on.ca:8180/ipt/resource.do?r=mamm

ROM Ornithology Collection - Non Passeriformes. Royal Ontario Museum. [Internet]. 2015. [Accessed 2015 Apr 18]. Available from: http://gbif.rom.on.ca:8180/ipt/resource.do?r=birdsnonpass

ROM Ornithology Collection - Passeriformes. Royal Ontario Museum. [Internet]. 2015. [Accessed 2015 Apr 18]. Available from: http://gbif.rom.on.ca:8180/ipt/resource.do?r=birdspass

ROM Palaeobiology - Vertebrate Fossils Collection - Mammalia. Royal Ontario Museum. [Internet]. 2015. [Accessed 2015 Apr 18]. Available from: http://gbif.rom.on.ca:8180/ipt/resource.do?r=vpfossils

ROM Palaeobiology - Vertebrate Fossils Collection - Non Mammalia. Royal Ontario Museum. [Internet]. 2015. [Accessed 2015 Apr 18]. Available from: http://gbif.rom.on.ca:8180/ipt/resource.do?r=vpfossils2

ROM Palaeobiology - Vertebrate Comparative Osteology Collection. Royal Ontario Museum. [Internet]. 2015. [Accessed 2015 Apr 18]. Available from: http://gbif.rom.on.ca:8180/ipt/resource.do?r=vposteology

Royal D. Suttkus Fish Collections. Tulane University, Biodiversity Research Institute. [Internet]. 2015. [Accessed 2015 Apr 18]. Available from: http://data.tubri.org/

SBMNH Vertebrate Collection. Santa Barbara Museum of Natural History. [Internet]. 2015. [Accessed 2015 Apr 18]. Available from: http://ipt.vertnet.org:8080/ipt/resource.do?r=sbmnh_verts

SDNHM Birds Collection. San Diego Natural History Museum. [Internet]. 2015. [Accessed 2015 Apr 18]. Available from: http://ipt.vertnet.org:8080/ipt/resource.do?r=sdnhm_birds

SMNS Herpetologie. Staatliches Museum für Naturkunde Stuttgart. [Internet]. 2015. [Accessed 2015 Apr 18]. Available from: http://ipt.vertnet.org:8080/ipt/resource.do?r=smns_herps

South Australian Museum Australia provider for OZCAM. South Australian Museum. [Internet]. 2015. [Accessed 2015 Apr 18]. Available from: http://collections.ala.org.au/public/show/dr346

Tasmanian Museum and Art Gallery provider for OZCAM. Tasmanian Museum and Art Gallery. [Internet]. 2015. [Accessed 2015 Apr 18]. Available from: http://collections.ala.org.au/public/show/dr347

TCWC Vertebrates. Biodiversity Research and Teaching Collections, Department of Wildlife and Fisheries Sciences, Texas A&M University. [Internet]. 2015. [Accessed 2015 Apr 18]. Available from: http://ipt.vertnet.org:8080/ipt/resource.do?r=tcwc_verts

The Fish Collection. Zoological Museum, Natural History Museum of Denmark. [Internet]. 2015. [Accessed 2015 Apr 18]. Available from: http://danbif.au.dk/ipt/resource.do?r=ds10

TNHC Herpetology Collection. The University of Texas at Austin - Texas Natural History Collections. [Internet]. 2015. [Accessed 2015 Apr 18]. Available from: http://ipt.vertnet.org:8080/ipt/resource.do?r=tnhc_herps

TNHC Ichthyology Collection (2013). Contributed by Hendrickson D. A. and A. E. Cohen.. Texas Natural History Collections, The University of Texas at Austin. [Internet]. 2015. [Accessed 2015 Apr 18]. Available from: http://ipt.vertnet.org:8080/ipt/resource.do?r=tnhc_fish

TTU Mammals Collection. Museum of Texas Tech University. [Internet]. 2015. [Accessed 2015 Apr 18]. Available from: http://ipt.vertnet.org:8080/ipt/resource.do?r=ttu_mammals

UAIC Ichthyological Collection. University of Alabama Biodiversity and Systematics. [Internet]. 2015. [Accessed 2015 Apr 18]. Available from: http://ipt.vertnet.org:8080/ipt/resource.do?r=uaic_fish

UAM Bird Collection (Arctos). University of Alaska Museum, University of Alaska Fairbanks. [Internet]. 2015. [Accessed 2015 Apr 18]. Available from: http://ipt.vertnet.org:8080/ipt/resource.do?r=uam_bird

UAM Earth Sciences Collection (Arctos). University of Alaska Museum, University of Alaska Fairbanks. [Internet]. 2015. [Accessed 2015 Apr 18]. Available from: http://ipt.vertnet.org:8080/ipt/resource.do?r=uam_es

UAM Fish Collection (Arctos). University of Alaska Museum, University of Alaska Fairbanks. [Internet]. 2015. [Accessed 2015 Apr 18]. Available from: http://ipt.vertnet.org:8080/ipt/resource.do?r=uam_fish

UCLA Donald R. Dickey Bird and Mammal Collection. University of California, Los Angeles. [Internet]. 2015. [Accessed 2015 Apr 18]. Available from: http://ipt.vertnet.org:8080/ipt/resource.do?r=ucla_birds_mammals

UCM Amphibians and Reptiles. University of Colorado Museum of Natural History. [Internet]. 2015. [Accessed 2015 Apr 18]. Available from: http://ipt.vertnet.org:8080/ipt/resource.do?r=ucm_herps

UCM Birds. University of Colorado Museum of Natural History. [Internet]. 2015. [Accessed 2015 Apr 18]. Available from: http://ipt.vertnet.org:8080/ipt/resource.do?r=ucm_birds

UCM Fishes. University of Colorado Museum of Natural History. [Internet]. 2015. [Accessed 2015 Apr 18]. Available from: http://ipt.vertnet.org:8080/ipt/resource.do?r=ucm_fish

UCM Mammals Collection. University of Colorado Museum of Natural History. [Internet]. 2015. [Accessed 2015 Apr 18]. Available from: http://ipt.vertnet.org:8080/ipt/resource.do?r=ucm_mammals

UMMZ Bird Collection. University of Michigan Museum of Zoology. [Internet]. 2015. [Accessed 2015 Apr 18]. Available from: http://ipt.vertnet.org:8080/ipt/resource.do?r=ummz_birds

UMMZ Herpetology Collection. University of Michigan Museum of Zoology. [Internet]. 2015. [Accessed 2015 Apr 18]. Available from: http://ipt.vertnet.org:8080/ipt/resource.do?r=ummz_herps

UMMZ Mammal Collection. University of Michigan Museum of Zoology. [Internet]. 2015. [Accessed 2015 Apr 18]. Available from: http://ipt.vertnet.org:8080/ipt/resource.do?r=ummz_mammals

UMZC Zoological Specimens. University Museum of Zoology Cambridge. [Internet]. 2015. [Accessed 2015 Apr 18]. Available from: http://ipt.vertnet.org:8080/ipt/resource.do?r=umzc_vertebrates

University of California Museum of Paleontology. University of California, Berkeley. [Internet]. 2015. [Accessed 2015 Apr 18]. Available from: http://bnhmipt.berkeley.edu/ipt/resource.do?r=ucmp

University of Kansas Bird Collection. KU Biodiversity Institute, University of Kansas. [Internet]. 2015. [Accessed 2015 Apr 18]. Available from: http://ipt.nhm.ku.edu/ipt/resource.do?r=kubi_ornithology

University of Kansas Bird Tissue Collection. KU Biodiversity Institute, University of Kansas. [Internet]. 2015. [Accessed 2015 Apr 18]. Available from: http://ipt.nhm.ku.edu/ipt/resource.do?r=kubi_ornithology_tissue

UTA Herpetology. University of Texas at Arlington Amphibian and Reptile Diversity Research Center. [Internet]. 2015. [Accessed 2015 Apr 18]. Available from: http://ipt.vertnet.org:8080/ipt/resource.do?r=uta_herps

UTEP Vertebrates. Laboratory for Environmental Biology, Centennial Museum, University of Texas at El Paso. [Internet]. 2015. [Accessed 2015 Apr 18]. Available from: http://ipt.vertnet.org:8080/ipt/resource.do?r=utep_verts

UWBM Mammalogy Collection. University of Washington Burke Museum. [Internet]. 2015. [Accessed 2015 Apr 18]. Available from: http://ipt.vertnet.org:8080/ipt/resource.do?r=uwbm_mammals

UWBM Ornithology Collection. University of Washington Burke Museum. [Internet]. 2015. [Accessed 2015 Apr 18]. Available from: http://ipt.vertnet.org:8080/ipt/resource.do?r=uwbm_birds

UWFC Ichthyology Collection. University of Washington Burke Museum. [Internet]. 2015. [Accessed 2015 Apr 18]. Available from: http://ipt.vertnet.org:8080/ipt/resource.do?r=uwfc_fish

UWYMV Bird Collection (Arctos). University of Wyoming Museum of Vertebrates. [Internet]. 2015. [Accessed 2015 Apr 18]. Available from: http://ipt.vertnet.org:8080/ipt/resource.do?r=uwymv_bird

UWYMV Mammal Collection (Arctos). University of Wyoming Museum of Vertebrates. [Internet]. 2015. [Accessed 2015 Apr 18]. Available from: http://ipt.vertnet.org:8080/ipt/resource.do?r=uwymv_mamm

Western Australian Museum provider for OZCAM. Western Australian Museum. [Internet]. 2015. [Accessed 2015 Apr 18]. Available from: http://collections.ala.org.au/public/show/dr348

WFVZ Bird Collections. Western Foundation of Vertebrate Zoology. [Internet]. 2015. [Accessed 2015 Apr 18]. Available from: http://ipt.vertnet.org:8080/ipt/resource.do?r=wfvz_birds

WNMU Fish Collection (Arctos). Western New Mexico University. [Internet]. 2015. [Accessed 2015 Apr 18]. Available from: http://ipt.vertnet.org:8080/ipt/resource.do?r=wnmu_fish

WNMU Mammal Collection (Arctos). Western New Mexico University. [Internet]. 2015. [Accessed 2015 Apr 18]. Available from: http://ipt.vertnet.org:8080/ipt/resource.do?r=wnmu_mamm

YPM Vertebrate Paleontology Division. Yale Peabody Museum. [Internet]. 2015. [Accessed 2015 Apr 18]. Available from: http://ipt.peabody.yale.edu/ipt/resource.do?r=ipt_vp

YPM Vertebrate Zoology Division - Herpetology. Yale Peabody Museum. [Internet]. 2015. [Accessed 2015 Apr 18]. Available from: http://ipt.peabody.yale.edu/ipt/resource.do?r=ipt_vz_her

YPM Vertebrate Zoology Division - Ichthyology. Yale Peabody Museum. [Internet]. 2015. [Accessed 2015 Apr 18]. Available from: http://ipt.peabody.yale.edu/ipt/resource.do?r=ipt_vz_ich

YPM Vertebrate Zoology Division - Mammalogy. Yale Peabody Museum. [Internet]. 2015. [Accessed 2015 Apr 18]. Available from: http://ipt.peabody.yale.edu/ipt/resource.do?r=ipt_vz_mam

YPM Vertebrate Zoology Division - Ornithology. Yale Peabody Museum. [Internet]. 2015. [Accessed 2015 Apr 18]. Available from: http://ipt.peabody.yale.edu/ipt/resource.do?r=ipt_vz_orn
