# Supplementary material for: A Standardized Reference Data Set for Vertebrate Taxon Name Resolution
Source: PLoS One. 2016 Jan 13;11(1):e0146894. doi: 10.1371/journal.pone.0146894 (PMC4711887; doi:10.1371/journal.pone.0146894)
Supplement: S1 Table — Fields are grouped into the four categories used in the study: Input, Convenience, Assessment and Output. Note that, among the assessment fields, sn-inf-missing only applies to scientificnameplus, and con-autherror, con-rnk and con-sgerror only apply to constructedscientificname since Darwin Core Standard does not by definition restrict the taxon rank to which scientificName can resolve to. In observations, we show to which type of issue we assigned each relevant assessment field. For evaluation of synonymy, comparisons were made between the contents of the Input and Convenience fields with the validCanonical name, and results were put into isSynonym field. For definitions of conceptual and format errors, see text. con-: referring to the field constructedscientificname, sn-: referring to the field scientificnameplus; DwC: Darwin Core Standard. (DOC) [file pone.0146894.s006.doc]

**S1 Table. Fields used to construct the Reference Data Set for Vertebrate Taxon Name Resolution.**

| **Field Group** | **Field** | **Definition** | **Observations** |
| --- | --- | --- | --- |
| Input | Genus | DwC genus field. | Verbatim |
| Input | Subgenus | DwC subgenus field. | Verbatim |
| Input | specificepithet | DwC specificEpithet field. | Verbatim |
| Input | infraspecificepithet | DwC infraspecificEpithet field. | Verbatim |
| Input | scientificnameauthorship | DwC scientificNameAuthorship field. | Verbatim |
| Input | scientificname | DwC scientificName field. | Verbatim |
| Convenience | **constructedscientificname**  (***con***) | The space-separated concatenation of DwC fields genus, subgenus, specificEpithet, infraspecificEpithet and scientificNameAuthorship. |  |
| Convenience | con-rank | The most specific taxon rank in the con field. |  |
| Convenience | **scientificnameplus** (***sn***) | The scientificName or the concatenation of scientificName with infraspecificEpithet when infraspecificEpithet is not null, genus is null, specificEpithet is null and an infraspecific epithet is not included in scientificName. |  |
| Convenience | sn-rank | The most specific rank in the scientificName field (not scientificnameplus). Added after resolution was done, for statistics purposes. |  |
| Assessment | con-auth, sn-auth | There is an author in the con or sn fields. |  |
| Assessment | con-sg, sn-sg | There is a subgenus in the con field. |  |
| Assessment | sn-inf-missing | There is an infraspecific epithet in the infraspecificEpithet field, but it is not included in the scientificname. | Error: Conceptual |
| Assessment | con-ms, sn-ms | There is a misspelling in the con or sn fields. | Error: Misspelling |
| Assessment | con-ab, sn-ab | There is a name abbreviation in the con or sn fields. | Error: Format |
| Assessment | con-authcap, sn-authcap | There is incorrect capitalization in the author in the con or sn fields. | Error: Format |
| Assessment | con-autherror | There is a scientific name authorship string in the constructedscientificname, but not from the scientificNameAuthorship input field. | Error: Conceptual |
| Assessment | con-cap, sn-cap | There is incorrect capitalization in the con or sn fields. | Error: Format |
| Assessment | con-cf, sn-cf | There is a cf identification qualifier in the con or sn fields. | Error: Conceptual |
| Assessment | con-enc, sn-enc | There is an encoding issue in the con or sn fields. | Error: Misspelling |
| Assessment | con-ex, sn-ex | There is something extra in the con or sn fields (distinct from the others set apart already). | Error: Conceptual |
| Assessment | con-hyb, sn-hyb | There is a hybrid formula in the con or sn fields. | Error: Conceptual |
| Assessment | con-inf, sn-inf | There is an infraspecific identification qualifier in the con or sn fields. | Error: Conceptual |
| Assessment | con-qu, sn-qu | There is a question mark identification qualifier in the con or sn fields. | Error: Conceptual |
| Assessment | con-rnk | There is an incorrect taxon rank in the con field. | Error: Conceptual |
| Assessment | con-sgerror | There is a subgenus in the constructedscientificname field, but not from the subgenus field. | Error: Conceptual |
| Assessment | con-sp, sn-sp | There is a species identification qualifier in the con or sn fields. | Error: Conceptual |
| Assessment | con-ws, sn-ws | There is extra whitespace in the con or sn fields. | Error: Format |
| Assessment | **hasIssue** | There is at least one issue (synonymy, misspelling, conceptual or format errors) in the name combination. | Overall validity of the name combination |
| Assessment | **isSynonym** | The name in intendedcanonical is a synonym of the one in validCanonical | Synonymy |
| Assessment | **hasMisspelling** | The name combination has a misspelling or encoding issue. | Overall prevalence of Misspelling |
| Assessment | **hasConceptualError** | The name combination has a conceptual error. | Overall prevalence of conceptual errors |
| Assessment | **hasFormatError** | The name combination has a format error. | Overall prevalence of format errors |
| Assessment | **con-valid** | The constructedscientificname is “valid”, “invalid” or “not applicable”. | Whole ***con*** assessment |
| Assessment | **sn-valid** | The scientificnameplus is “valid”, “invalid” or “not applicable”. | Whole ***sn*** assessment |
| Assessment | **intendedcanonical** | The interpretation of the correct (not necessarily valid) canonical scientific name based on the input fields, after correcting for errors. |  |
| Assessment | **intendedtaxonrank** | The taxon rank of the intendedcanonical name. |  |
| Output | **validCanonical** | The canonical form (monomial, binomial, or trinomial, no authorship) of the scientific name based on the input fields and the external sources consulted. |  |
| Output | **validtaxonrank** | The taxon rank of the the validCanonical name. |  |
| Output | **validSource** | The name(s) of the source(s) used to determine the name in the validCanonical field. |  |
| Output | **validURL** | URL of the source(s) used to determine the name in the validCanonical field. |  |
| Output | **sourcedate** | The date on which the validSource was consulted. |  |
| Output | Comment | Anything useful or interesting about the resolution of the name combination. |  |

Fields are grouped into the four categories used in the study: Input, Convenience, Assessment and Output. Note that, among the assessment fields, **sn-inf-missing** only applies to **scientificnameplus**, and **con-autherror**, **con-rnk** and **con-sgerror** only apply to **constructedscientificname** since Darwin Core Standard does not by definition restrict the taxon rank to which **scientificName** can resolve to. In observations, we show to which type of issue we assigned each relevant assessment field. For evaluation of synonymy, comparisons were made between the contents of the Input and Convenience fields with the **validCanonical** name, and results were put into **isSynonym** field. For definitions of conceptual and format errors, see text. **con-**: referring to the field **constructedscientificname**, **sn-**: referring to the field **scientificnameplus**; DwC: Darwin Core Standard.
