# Supplementary material for: A Standardized Reference Data Set for Vertebrate Taxon Name Resolution
Source: PLoS One. 2016 Jan 13;11(1):e0146894. doi: 10.1371/journal.pone.0146894 (PMC4711887; doi:10.1371/journal.pone.0146894)
Supplement: S2 Table — Number of name combinations for which a field or characteristic of the name combination matched a given condition. Numbers outside of parentheses are for the 991 name combinations for which there was no disagreement between the assessment of the two researchers. Numbers in parentheses are the counts for the results that had multiple opinions on valid names. For definitions of fields, see S1 Table. * Classes included in “Fishes” clade for analysis of drivers of issues. (DOC) [file pone.0146894.s007.doc]

**S2 Table. Detailed assessment of the 1000 name combinations.**

| **Field / Characteristic** | **Condition** | **Name Combinations** |
| --- | --- | --- |
| con-ms | TRUE | 114 |
| sn-ms | TRUE | 59 |
| con-ms or sn-ms | TRUE | 128 |
| con-sp | TRUE | 33 |
| sn-sp | TRUE | 48 |
| con-sp or sn-sp | TRUE | 57 |
| con-inf | TRUE | 7 |
| sn-inf | TRUE | 4 |
| con-inf or sn-inf | TRUE | 8 |
| sn-inf-missing | TRUE | 6 |
| con-ws | TRUE | 1 |
| sn-ws | TRUE | 14 (1/1) |
| con-ws or sn-ws | TRUE | 15 |
| con-cap | TRUE | 41 (2/2) |
| sn-cap | TRUE | 9 |
| con-cap or sn-cap | TRUE | 43 |
| con-sg | TRUE | 6 |
| sn-sg | TRUE | 5 |
| con-sg or sn-sg | TRUE | 10 |
| con-sg-error | TRUE | 1 |
| con-rnk | TRUE | 3 |
| con-auth | TRUE | 203 (2/2) |
| sn-auth | TRUE | 66 |
| con-auth or sn-auth | TRUE | 225 |
| con-autherror | TRUE | 1 (1/1) |
| con-authcap | TRUE | 27 |
| sn-authcap | TRUE | 26 |
| con-authcap or sn-authcap | TRUE | 27 |
| con-hyb | TRUE | 2 |
| sn-hyb | TRUE | 2 |
| con-hyb or sn-hyb | TRUE | 3 |
| con-cf | TRUE | 5 |
| sn-cf | TRUE | 6 |
| con-cf or sn-cf | TRUE | 8 |
| con-qu | TRUE | 15 |
| sn-qu | TRUE | 13 |
| con-qu or sn-qu | TRUE | 19 |
| con-ab | TRUE | 3 |
| sn-ab | TRUE | 1 |
| con-ab or sn-ab | TRUE | 4 |
| con-ex | TRUE | 40 |
| sn-ex | TRUE | 2 |
| con-ex or sn-ex | TRUE | 31 |
| con-enc | TRUE | 1 |
| sn-enc | TRUE | 0 |
| con-enc or sn-enc | TRUE | 1 |
| con-valid | valid | 397 (2/2) |
|  | invalid | 518 (6/6) |
|  | not applicable | 76 (1/1) |
| sn-valid | valid | 336 (1/2) |
|  | invalid | 324 (4/3) |
|  | not applicable | 331 (4/4) |
| con-valid or sn-valid | valid | 475 (2/3) |
|  | invalid | 590 (8/7) |
| con-valid and sn-valid | valid | 258 (1/1) |
|  | invalid | 252 (2/2) |
| con-rank | species | 559 (2/2) |
|  | subspecies | 246 (5/5) |
|  | genus | 80 (1/1) |
|  | subgenus | 4 |
|  | family | 2 |
|  | tribe | 1 |
|  | null | 99 (1/1) |
| sn-rank | species | 432 (2/2) |
|  | subspecies | 154 (3/3) |
|  | genus | 68 |
|  | family | 5 |
|  | tribe | 1 |
|  | null | 331 (4/4) |
| con-rank same as sn-rank | TRUE | 523 (3/3) |
| con-rank not same as sn-rank | TRUE | 468 (6/6) |
| dwcsn-rank | species | 438 (2/2) |
|  | subspecies | 148 (3/3) |
|  | genus | 68 |
|  | family | 5 |
|  | tribe | 1 |
|  | null | 331 (4/4) |
| validtaxonrank | species | 651 (5/7) |
|  | subspecies | 208 (4/2) |
|  | genus | 90 |
|  | family | 7 |
|  | tribe | 1 |
|  | null | 34 |
| scientificName | =constructedscientificname  (plus secondary opinions) | 307 (2/2) |
|  | !=constructedscientificname  (plus secondary opinions) | 297 (2/2) |
|  | not null while constructedscientificname is null (plus secondary opinions) | 56 (1/1) |
|  | null (plus secondary opinions) | 331 (4/4) |
| intendedcanonical | =validCanonical  (plus secondary opinions) | 668 (2/3) |
|  | !=validCanonical  (plus secondary opinions) | 290 (7/6) |
|  | not null while validCanonical is null | 11 (0/0) |
|  | null | 22 (0/0) |
| constructedscientificname | =validCanonical  (plus secondary opinions) | 39 (2/1) |
|  | !=validCanonical  (plus secondary opinions) | 589 (6/7) |
|  | not null while validCanonical is null | 31 (0/0) |
|  | null while validCanonical is not null  (plus secondary opinions) | 54 (1/1) |
|  | null while validCanonical is null | 2 (0/0) |
| scientificnameplus | =validCanonical  (plus secondary opinions) | 305 (1/2) |
|  | !=validCanonical  (plus secondary opinions) | 333 (4/3) |
|  | not null while validCanonical is null  (plus secondary opinions) | 22 (0/0) |
|  | null while validCanonical is not null  (plus secondary opinions) | 320 (4/4) |
|  | null while validCanonical is null | 11 (0/0) |
| class | Aves | 349 (5/5) |
|  | Actinopterygii* | 323 (2/2) |
|  | Mammalia | 151 (2/2) |
|  | Reptilia | 79 |
|  | Amphibia | 57 |
|  | Elasmobranchii* | 19 |
|  | Conodonta* | 5 |
|  | Holocephali* | 3 |
|  | Cephalaspidomorphi* | 2 |
|  | Sarcopterygii* | 1 |
|  | Placodermi* | 1 |
|  | Myxini* | 1 |

Number of name combinations for which a field or characteristic of the name combination matched a given condition. Numbers outside of parentheses are for the 991 name combinations for which there was no disagreement between the assessment of the two researchers. Numbers in parentheses are the counts for the results that had multiple opinions on valid names. For definitions of fields, see Table 1S.

* Classes included in “Fishes” clade for analysis of drivers of issues.
