# Supplementary material for: A Standardized Reference Data Set for Vertebrate Taxon Name Resolution
Source: PLoS One. 2016 Jan 13;11(1):e0146894. doi: 10.1371/journal.pone.0146894 (PMC4711887; doi:10.1371/journal.pone.0146894)
Supplement: S3 Table — Number of name combinations for which distinct taxonomic name sources provided the name given in validCanonical. We do not include 30 sources that were only consulted once. Some name combinations were checked utilizing multiple sources, and in some cases secondary sources used for confirmation were not always captured by the vetter. (DOC) [file pone.0146894.s008.doc]

**S3 Table. Taxonomic Name Sources Consulted.**

| **validSource** | **Name combinations** |
| --- | --- |
| Avibase | 268 |
| EN Wikipedia | 192 |
| FishBase | 183 |
| ITIS | 104 |
| WoRMS | 62 |
| Reptile Database | 58 |
| IUCN Redlist | 28 |
| Mammal Species of the World 3rd Edition | 21 |
| AmphibiaWeb | 20 |
| Fossilworks | 19 |
| Paleobiology Database | 13 |
| Mammals' Planet | 8 |
| Amphibian Species of the World 6.0 | 7 |
| ES Wikipedia | 6 |
| SV Wikipedia | 5 |
| Mammal Species of the World Vol. 1 | 4 |
| Handbook of the Birds of the World Alive | 4 |
| PL Wikipedia | 4 |
| FishWisePro | 2 |
| FR Wikipedia | 2 |

Number of name combinations for which distinct taxonomic name sources provided the name given in validCanonical. We do not include 30 sources that were only consulted once. Some name combinations were checked utilizing multiple sources, and in some cases secondary sources used for confirmation were not always captured by the vetter.
